# Evolutionary Ecology of Natural Comammox *Nitrospira* Populations

**DOI:** 10.1128/msystems.01139-21

**Published:** 2022-01-11

**Authors:** Alejandro Palomo, Arnaud Dechesne, Otto X. Cordero, Barth F. Smets

**Affiliations:** a Department of Environmental Engineering, Technical University of Denmark, Kongens Lyngby, Denmark; b Ralph M. Parsons Laboratory for Environmental Science and Engineering, Department of Civil and Environmental Engineering, Massachusetts Institute of Technology, Cambridge, Massachusetts, USA; University of Illinois at Urbana-Champaign

**Keywords:** comammox, evolutionary ecology, microdiversity, nitrification, selection

## Abstract

Microbes commonly exist in diverse and complex communities where species interact, and their genomic repertoires evolve over time. Our understanding of species interaction and evolution has increased during the last decades, but most studies of evolutionary dynamics are based on single species in isolation or in experimental systems composed of few interacting species. Here, we use the microbial ecosystem found in groundwater-fed sand filter as a model to avoid this limitation. In these open systems, diverse microbial communities experience relatively stable conditions, and the coupling between chemical and biological processes is generally well defined. Metagenomic analysis of 12 sand filters communities revealed systematic co-occurrence of at least five comammox *Nitrospira* species, likely promoted by low ammonium concentrations. These *Nitrospira* species showed intrapopulation sequence diversity, although possible clonal expansion was detected in a few abundant local comammox populations. *Nitrospira* species showed low homologous recombination and strong purifying selection, the latter process being especially strong in genes essential in energy metabolism. Positive selection was detected for genes related to resistance to foreign DNA and phages. We found that, compared to other habitats, groundwater-fed sand filters impose strong purifying selection and low recombination on comammox *Nitrospira* populations. These results suggest that evolutionary processes are more affected by habitat type than by species identity. Together, this study improves our understanding of species interaction and evolution in complex microbial communities and sheds light on the environmental dependency of evolutionary processes.

**IMPORTANCE** Microbial species interact with each other and their environment (ecological processes) and undergo changes in their genomic repertoire over time (evolutionary processes). How these two classes of processes interact is largely unknown, especially for complex communities, as most studies of microbial evolutionary dynamics consider single species in isolation or a few interacting species in simplified experimental systems. In this study, these limitations are circumvented by examining the microbial communities found in stable and well-described groundwater-fed sand filters. Combining metagenomics and strain-level analyses, we identified the microbial interactions and evolutionary processes affecting comammox *Nitrospira*, a recently discovered bacterial type capable of performing the whole nitrification process. We found that abundant and co-occurrent *Nitrospira* populations in groundwater-fed sand filters are characterized by low recombination and strong purifying selection. In addition, by comparing these observations with those obtained from *Nitrospira* species inhabiting other environments, we revealed that evolutionary processes are more affected by habitat type than by species identity.

## INTRODUCTION

Microorganisms dominate the tree of life based on species diversity, and they play an essential role in all global biogeochemical cycles. Microbial species interact with each other and with the environment (ecological processes) and also undergo changes in their genomic repertoire over time (evolutionary processes). Yet the interaction between ecological and evolutionary processes is largely unknown, especially for complex open communities. For many years, most studies of microbial communities in open, complex environments have focused on ecological aspects, as it was believed that evolutionary changes happen at a much larger timescale (1). However, in recent years, with the development of population genomics analysis, researchers have started to jointly investigate ecological and evolutionary processes. Yet most studies of evolutionary dynamics remain based on single species in isolation (2) or on experimental systems composed of only a few interacting species (3). While these analyses have helped to understand some aspects of evolutionary patterns, they have limitations because they lack many characteristics of true natural populations (e.g., spatial structure, existence of microdiversity, predation, immigration). On the other hand, observing populations in the wild also has limitations because the conditions vary with little control (hence, uncontrolled variation in population size, selection regime) and because the typically unknown ecophysiology of retrieved genomes makes it difficult to interpret the observed patterns. Therefore, studying well-defined model microbial ecosystems can help to understand ecological and evolutionary processes in microbial communities (4).

Rapid sand filters (RSF), widely used to produce drinking water from groundwater, are useful model systems. They are characterized by stable conditions, including active growth, primarily driven by the oxidation of ammonia, methane, and other inorganic compounds present at low concentrations in the influent water, large populations (10^9^ to 10^10^ cells/g), significant mixing (due to backwashing), continuous but limited immigration from prokaryotes in the influent water, and no dispersal between separate sand filters (resulting in allopatric populations) (5–8). In addition, the microbial communities inhabiting these systems, which are usually stable across time (9), have been broadly described, showing a general dominance of complete ammonia oxidizers (comammox) (6, 10, 11). These recently discovered microorganisms are expected to have a relatively simple ecology (due to their chemolithoautotrophic metabolism) (12), yet are poorly studied in terms of what drives their diversity, distribution, and evolution. Furthermore, as comammox bacteria occur in RSF as coexisting populations (10, 13), RSF offer an opportunity for resolving fine-scale genomic heterogeneity within closely related strains and investigating if they show similar patterns in evolutionary processes (such as selection or recombination).

Of particular interest is to determine to what extent the evolutionary processes that drive the diversification of comammox *Nitrospira* are dependent on their environment, as opposed to intrinsic properties of the species. The environmental dependency of microbial evolution has been investigated from different perspectives. Several studies have focused on genome signature variations (GC, tetranucleotide signatures, codon usage, purine-pyrimidine ratio) associated with different environments (reviewed in Dutta and Paul [14]). Others have studied bacterial adaptation to shifting environments (15) or have targeted a specific evolutionary process across several lifestyles (e.g., homologous recombination [16] or selection [17]). Most of these studies, however, considered different species living in different environments or closely related species with a different lifestyle (i.e., free-living organisms versus pathogens). Yet little is known about ongoing evolutionary processes for species belonging to the same lineage with presumed similar physiology inhabiting different open environments. In this study, taking advantage of the multiple comammox species present in several groundwater-fed RSF, we thoroughly investigated evolutionary processes in this environment and compared these observations with those in comammox species inhabiting other environments.

## RESULTS AND DISCUSSION

**FIG 1:**
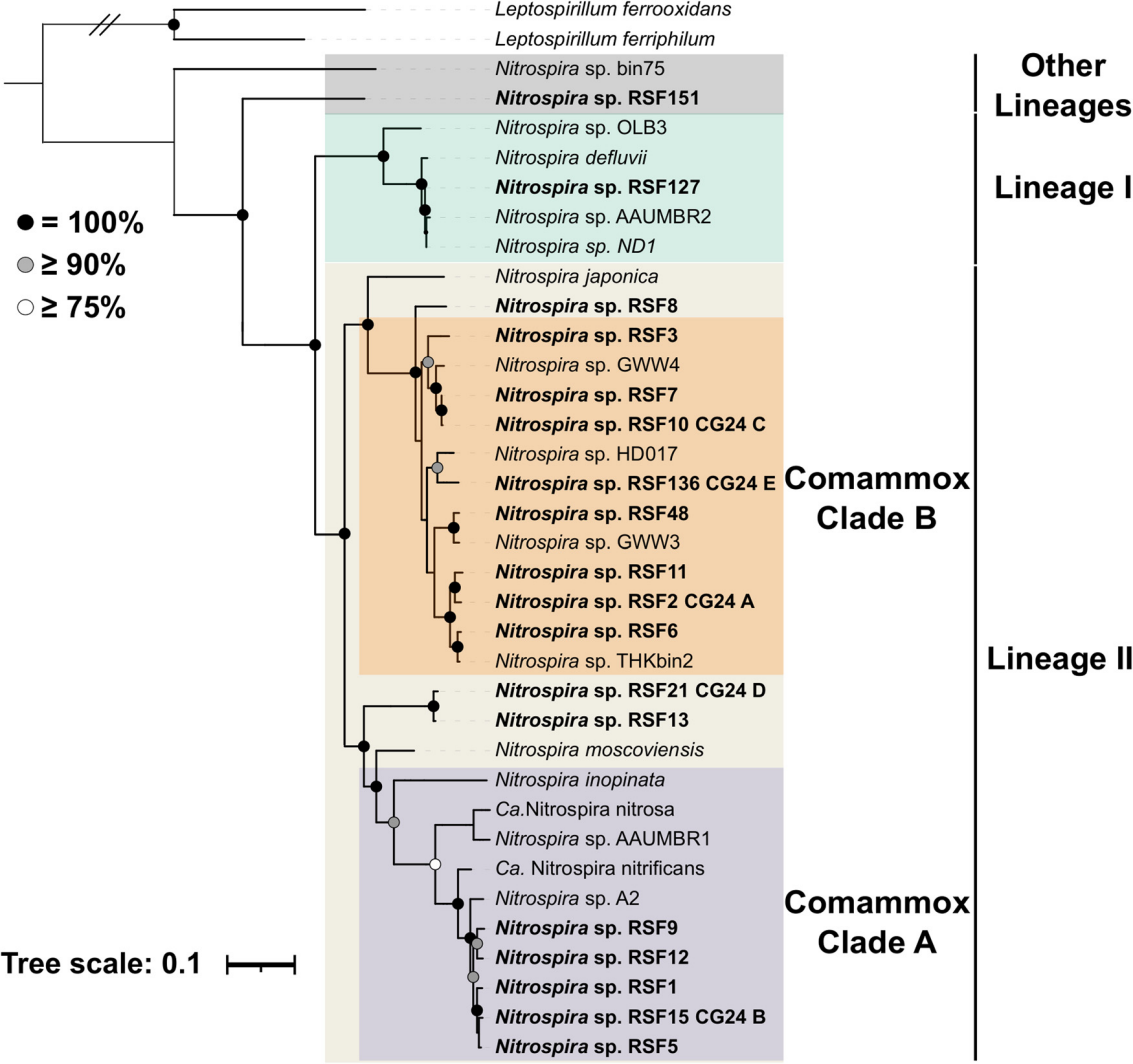
Phylogenomic affiliation of *Nitrospira* MAGs retrieved from 12 waterworks. A phylogenetic tree was built based on the concatenation of 120 proteins. *Nitrospira* MAGs retrieved in this study are highlighted in boldface. Lineages and sublineages are shown with colors (lineage I, green; lineage II, light brown; other lineages, gray; comammox clade A purple; comammox clade B, orange). *Leptospirillum* was used to root the tree. The strength of support for internal nodes as assessed by bootstrap replicates is indicated as colored circles (top left legend).

We examined ecological and evolutionary patterns within comammox-dominated bacterial communities inhabiting groundwater-fed rapid sand filters. To that end, we retrieved metagenome-assembled genomes (MAGs) from 12 similarly operated waterworks in Denmark, using a combination of automatic and manual binning, followed by several refinement steps to improve the bin quality (Fig. S1 in the supplemental material). To remove redundancy, a single representative was selected for each set of genomes that shared an average nucleotide identity (ANI) greater than 99%. This resulted in a total of 189 MAGs (genome completeness, 83.9% ± 13.6; contamination, 1.9% ± 1.4) (Table S1 at https://figshare.com/articles/dataset/Table_S1/17099123), 18 of them classified as *Nitrospira* spp. (completeness, 89.1% ± 9.2; contamination, 2.5% ± 1.1) (Table S2 at https://figshare.com/articles/dataset/Table_S2/17099138). These *Nitrospira* MAGs spanned 16 putative species (further on simply referred to as “species”) using a threshold average nucleotide identity (ANI) of ≥95% (18–20). The phylogenomic analysis placed 1 *Nitrospira* species into lineage I and 14 into lineage II, and another 1 was not assigned to any previously described lineage due to the lack of similar reference genomes (Fig. 1). Of the 16 *Nitrospira* species, 12 were classified as comammox *Nitrospira* (5 clade A and 7 clade B) (Fig. 1). As expected, the genomes classified as comammox *Nitrospira* also contained genes essential for complete ammonia oxidation, such as those of the ammonia monooxygenase (AMO) and hydroxylamine dehydrogenase (HAO) operons. Further description of the *Nitrospira* MAGs can be found elsewhere (21).

10.1128/mSystems.01139-21.1 FIG S1 Implemented workflow for the MAGs recovery from 12 Danish groundwater-fed rapid sand filters. The final genome quality improvement performed with MeDuSa was only applied on the 18 *Nitrospira* MAGs. The numbers in the binning algorithm boxes indicate the minimum contig size considered for the binning step. For MetaBAT, SupB20 and SPpB20 indicate “superspecific” and “verysensitive” modes, respectively. Download FIG S1, PDF file, 0.2 MB. Copyright © 2022 Palomo et al. 2022 Palomo et al. https://creativecommons.org/licenses/by/4.0/ This content is distributed under the terms of the Creative Commons Attribution 4.0 International license.

**FIG 2:**
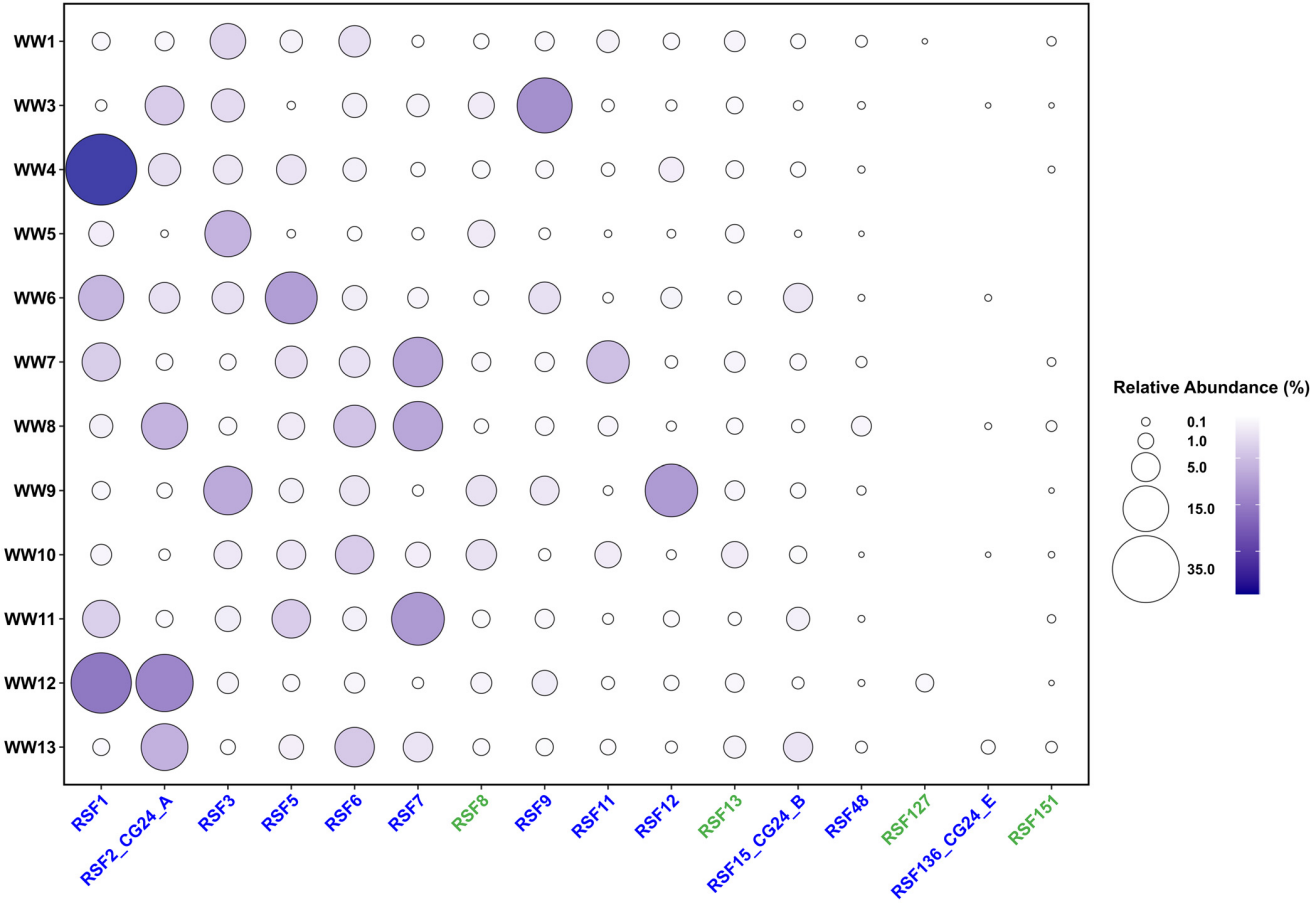
Abundance of *Nitrospira* species across 12 waterworks. Relative abundance (related to the total community) of 16 *Nitrospira* species in 12 waterworks. Comammox and canonical *Nitrospira* species are denoted in blue and green, respectively. Only relative abundances above 0.005% are considered.

*Nitrospira* species comprised a large proportion of the microbial communities of the waterworks (27 to 70%), and comammox represented a large fraction of *Nitrospira* spp. (76 to 98%) (Fig. 2 and Table S3 at https://figshare.com/articles/dataset/Table_S3/17099141). For most of the RSFs, the variation in the abundance of the different *Nitrospira* MAGs between duplicate samples was low (Fig. S2), suggesting a high spatial homogeneity in the top layer of the filters, probably due to the frequent mixing caused by backwash, and, hence, supporting the representativeness of our sampling. Multiple *Nitrospira* species (at least 5; 10 on average) co-occurred in all the waterworks (Fig. 2). However, there was no consistent dominance pattern. In some cases, a single species, but not always the same, dominated (waterworks 4 [WW4] and WW5), while in others, two (WW9 and WW12) or more species had similar high abundance (Fig. 2). The chemical characteristics of the water explained 57% of the variance in *Nitrospira* composition (permutation test, *P* < 0.001) (Table S4 at https://figshare.com/articles/dataset/Table_S4/17099150), suggesting that water chemistry is a strong filter for the assembly of these nitrifying communities. Among the measured water constituents, the influent ammonium concentration best explained *Nitrospira* distribution (explained 18%; permutation test, *P* = 0.02) (Table S4 at https://figshare.com/articles/dataset/Table_S4/17099150). Higher comammox species richness was detected in waterworks treating lower ammonium concentration (Fig. S3) (*R*^2^ = 0.54; *P* < 0.01). Canonical *Nitrospira* and canonical ammonia oxidizers were more abundant in waterworks receiving influent with higher ammonium (Fig. S4). These observations are in line with the prediction that higher ammonium concentration favors the division of labor between canonical ammonia and nitrite oxidizer (22). Nevertheless, we observed that one comammox species (RSF3) seemed to cope with slightly higher ammonium concentrations as well (Fig. S4). Interestingly, besides the mentioned RSF3, the other comammox *Nitrospira* species tended to cluster with other members of the same clade (clades are separated based on the phylogeny of ammonia monooxygenase subunit) (Fig. S4). The distribution pattern of *Nitrospira* species across the waterworks was not related to their geographic distance (Mantel test; *r* statistic, 0.08; significance > 0.05).

10.1128/mSystems.01139-21.2 FIG S2 Average relative abundance (*n* = 2) of 16 *Nitrospira* species in 12 waterworks. Comammox and canonical *Nitrospira* species are denoted in blue and green, respectively. Download FIG S2, PDF file, 0.4 MB. Copyright © 2022 Palomo et al. 2022 Palomo et al. https://creativecommons.org/licenses/by/4.0/ This content is distributed under the terms of the Creative Commons Attribution 4.0 International license.

10.1128/mSystems.01139-21.3 FIG S3 Relationship between the ammonium concentration and the comammox species richness in each waterworks (number of species with abundances >0.5%). Blue line shows the linear regression, with shadowed region indicating 95% confidence intervals for the slope (*P* value for *R*^2^ < 0.01). Download FIG S3, PDF file, 0.1 MB. Copyright © 2022 Palomo et al. 2022 Palomo et al. https://creativecommons.org/licenses/by/4.0/ This content is distributed under the terms of the Creative Commons Attribution 4.0 International license.

10.1128/mSystems.01139-21.4 FIG S4 Redundancy analysis (RDA) of the relationship between water chemistry and the species abundance in 12 waterworks. Arrows show the association of individual species (different colors) and water chemistry variables (blue arrows) with each axis. Pink, comammox clade A; orange, comammox clade B; green, canonical *Nitrospira*; brown, β-canonical AOB. Download FIG S4, PDF file, 0.5 MB. Copyright © 2022 Palomo et al. 2022 Palomo et al. https://creativecommons.org/licenses/by/4.0/ This content is distributed under the terms of the Creative Commons Attribution 4.0 International license.

Significant correlation between the abundances of the comammox species was rare; only a few strong positive correlations were detected (RSF5 and RSF15_CG24_B [ρ = 0.84]; RSF6 and RSF11 [ρ = 0.84]), while no significant negative correlations were observed (Fig. S5). This absence of obvious competitive exclusion pattern, together with the aforementioned description of the coexistence of multiple comammox species, suggests that these microorganisms exploit slightly different niches. An explanation might be that the co-occurrent comammox *Nitrospira* species had different ammonia affinities, and they would occupy different sites in the highly porous filter material (23). In fact, ammonium concentration was the variable that better explained the *Nitrospira* distribution in the studied waterworks. Although little is known about the range of ammonia affinities in comammox *Nitrospira*, as this trait has only been measured in two species (24, 25), other ammonia oxidizers have ammonia affinities spanning several orders of magnitude (26). Likewise, canonical *Nitrospira* species affiliated with different lineages have shown niche differentiation based on nitrite concentration (27, 28). Another explanation could be a dissimilar use of alternative metabolisms. It has been documented that some *Nitrospira* species can use hydrogen as energy source (29) and formate as carbon and/or energy source (depending on the *Nitrospira* type) (28). While some of the comammox genomes recovered from the waterworks encode genes for formate oxidation, others harbor genes putatively involved in hydrogen metabolism (Table S5 at https://figshare.com/articles/dataset/Table_S5/17099153). Another feature that may contribute to niche differentiation is the distinct N assimilation capacity, as the comammox genomes contain transporters with different ammonium affinity and uptake capacity (13) (Table S5 at https://figshare.com/articles/dataset/Table_S5/17099153). Besides this, each comammox *Nitrospira* species contain a large number of unique genes clusters (>250 on average) (21). Although the function of most of them is unknown, these unique genes might promote ecological variation. Traits such as chemotactic strategies, attachment to particles strategies, secondary metabolism, or defense against predation have been proposed to explain this phenomenon in other coexisting microorganisms (reviewed in Louca et al. [30]).

10.1128/mSystems.01139-21.5 FIG S5 (A) Correlogram showing proportionality for centered log-transformed abundances of the *Nitrospira* and AOB species across the 12 waterworks. Color indicates whether the correlation is positive (purple) or negative (brown). Size and darkness of the circles indicate the strength of the proportionality, with stronger proportionality being larger and darker than weaker ones. Asterisk indicates significant proportionality (FDR < 0.05). (B) Network analysis revealing the co-occurrence patterns among species present in the studied waterworks. Each node represents a species and is colored according to species type (lineage II canonical *Nitrospira*, green; comammox, blue; AOB, red; other bacteria, yellow). A connection represents a strong proportionality (*ρ > *0.55 and FDR < 0.05). The size of each node is proportional to the average species log-transformed abundance. Only nodes connected with *Nitrospira* species are shown. The MAG identity of the *Nitrospira* species is displayed with a number on top of the node (e.g., 1 stands for the species RSF1). Download FIG S5, PDF file, 0.6 MB. Copyright © 2022 Palomo et al. 2022 Palomo et al. https://creativecommons.org/licenses/by/4.0/ This content is distributed under the terms of the Creative Commons Attribution 4.0 International license.

### Microdiversity within *Nitrospira* species.

Strain-level analysis across the waterworks revealed that the *Nitrospira* populations contained intraspecies sequence diversity. We exploited the shotgun metagenomic data to perform strain-level analyses on the 12 most abundant *Nitrospira* species (genome completeness, 92.4% ± 5.5; contamination, 2.7% ± 0.8) based on single nucleotide polymorphisms (SNPs). The number of SNPs/Mbp in the populations across the waterworks ranged from 14,437 to 45,664 (Table S6 at https://figshare.com/articles/dataset/Table_S6/17099159). Looking into the populations at the local scale (species within waterworks), the number of SNPs/Mbp ranged from 249 to 37,663 (Table S7 at https://figshare.com/articles/dataset/Table_S7/17099171).

**FIG 3:**
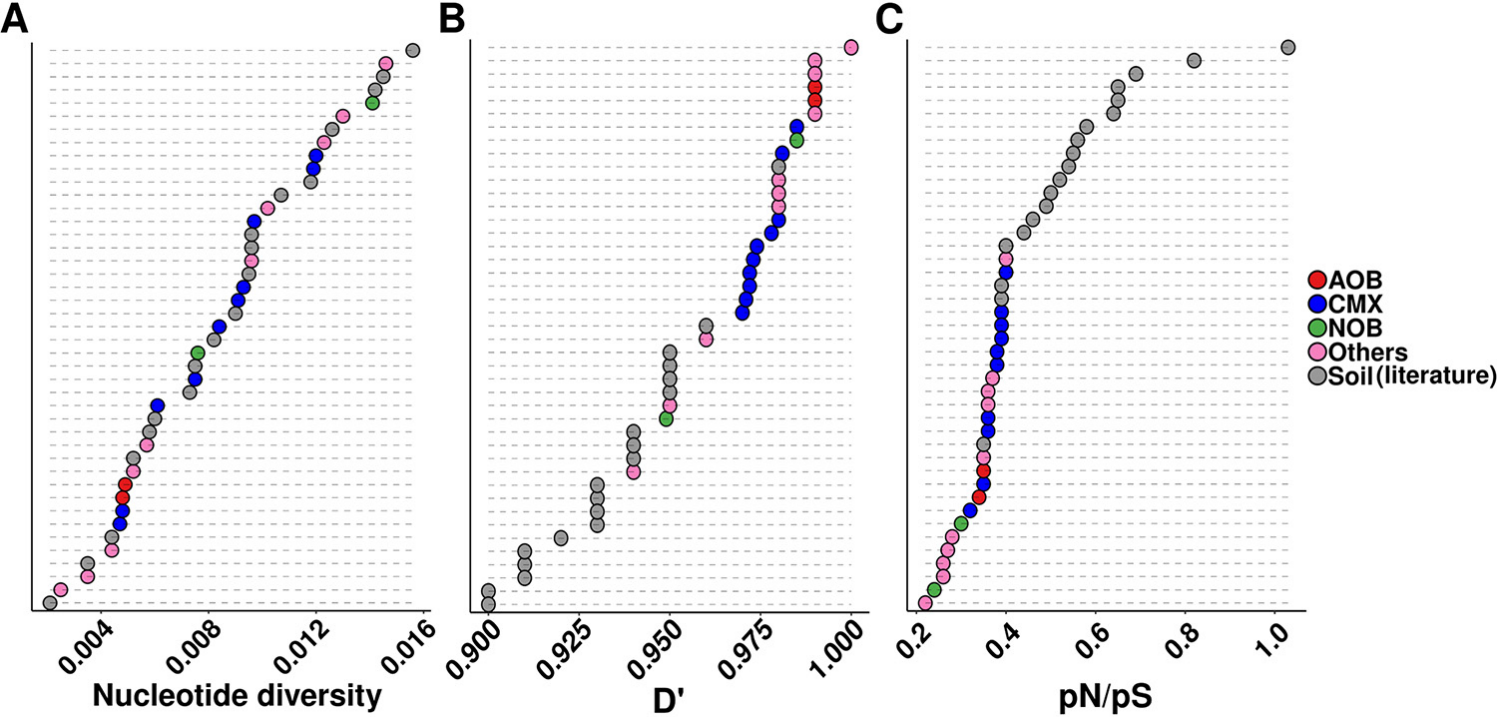
Evolutionary metrics of *Nitrospira* populations across 12 waterworks compared to other organisms and locations. (A) Nucleotide diversity (π) of the most abundant bacterial populations across 12 waterworks and the most abundant bacterial populations across a grassland meadow (denoted as “soil”) (38). (B) Homologous recombination (*D*′) of most abundant bacterial populations across 12 waterworks and most abundant bacterial populations across grassland meadow. (C) Selection (pN/pS ratio) of most abundant bacterial populations across 12 waterworks and most abundant bacterial populations across grassland meadow. AOB, ammonia-oxidizing bacteria; CMX, comammox; NOB, nitrite oxidizing bacteria; others, nonnitrifying abundant bacteria present in the waterworks. The data reported in this figure (including the MAGs ID) can be found at https://figshare.com/articles/dataset/Table_S6/17099159.

We observed a wide range of microdiversity (measured as nucleotide diversity [π]) among species (Fig. 3A): canonical *Nitrospira* RSF8 was the most diverse species, with three times more nucleotide diversity than the less diverse *Nitrospira* species of our study (RSF1 and RSF12) (Fig. 3A). Depending on the *Nitrospira* population, we detected instances of both homogeneous microdiversity across the waterworks (e.g., RSF5 and RSF8), as well as a high microdiversity variation depending on the waterworks (e.g., RSF1, RSF9, and RSF11) (Fig. S6). Based on our observations of high species-level comammox *Nitrospira* diversity at low ammonium concentration, we hypothesized that such conditions also promote high microdiversity. However, this was not the case, as the correlations of microdiversity with ammonium concentration or with comammox species richness were not significant for any species (*P* > 0.05). This suggests different drivers for interspecies versus within-species diversities.

10.1128/mSystems.01139-21.6 FIG S6 Boxplot showing the nucleotide diversity values for each *Nitrospira* species in the studied waterworks. Only species with more than two data points are shown. Download FIG S6, PDF file, 0.2 MB. Copyright © 2022 Palomo et al. 2022 Palomo et al. https://creativecommons.org/licenses/by/4.0/ This content is distributed under the terms of the Creative Commons Attribution 4.0 International license.

While all species showed significant microdiversity across waterworks, we detected a few highly abundant comammox populations with almost no microdiversity at the local scale (e.g., comammox *Nitrospira* RSF1 and RSF12 in WW4 and WW9, respectively) (Fig. S7), which suggests local clonal expansions of these comammox populations. Along the same line, the analysis of major allele frequencies of common SNPs revealed that only a fraction of the within-species variants diversity is found locally, and in some cases, distinct single variants dominated different waterworks (e.g., comammox RSF3 in WW10B versus WW5) (Fig. S8). These results contrast with species-level observations, where all the diversity was represented in each waterworks (as they all contain most of the *Nitrospira* species) (Fig. 2).

10.1128/mSystems.01139-21.7 FIG S7 Relationship between nucleotide diversity and coverage for each *Nitrospira* population in each waterworks. The mean nucleotide diversity and the mean coverage across species in each waterworks are shown. Local populations with high coverage and low nucleotide diversity are colored in red. (linear regression; *R*^2^ = 0.07; *P* > 0.01). Download FIG S7, PDF file, 0.2 MB. Copyright © 2022 Palomo et al. 2022 Palomo et al. https://creativecommons.org/licenses/by/4.0/ This content is distributed under the terms of the Creative Commons Attribution 4.0 International license.

10.1128/mSystems.01139-21.8 FIG S8 Heatmap of the major allele frequency for common SNPs (rows) in the waterworks populations (columns; only populations with coverage >5 are included) of the different *Nitrospira* species. Color represents the frequency of major allele of each SNP from 0 (red) to 1 (blue). Dendrograms group populations based on the frequency similarity. The order of the eights plots from left to right and top to bottom corresponds to the following species: RSF1, RSF2, RSF3, RSF5, RSF6, RSF7, RSF8, and RSF13 (this analysis only includes the RSF species with a coverage of >5 in at least 10 samples). Download FIG S8, PDF file, 1.2 MB. Copyright © 2022 Palomo et al. 2022 Palomo et al. https://creativecommons.org/licenses/by/4.0/ This content is distributed under the terms of the Creative Commons Attribution 4.0 International license.

Similar to what we observed at the species level, there was no significant correlation between similarity in subspecies composition and the geographic distance of the waterworks, with the exceptions of *Nitrospira* sp. RSF2 (*P* < 0.01) and *Nitrospira* sp. RSF8 (*P* < 0.05) (Fig. S9). However, we observed an interwaterworks organization of the genetic structure at most loci across the studied genomes, indicating that the *Nitrospira* populations were more similar within than between waterworks. For each gene, we calculated pairwise fixation indexes (*F*_ST_) to measure differences in allele frequencies between populations of the same species found in two distinct waterworks. The mean gene F*_ST_* values were ≥0.15 for all *Nitrospira* species (Fig. S10), the most extreme case being RSF5 (*F*_ST_ > 0.4), which suggests a great genetic differentiation among the populations (Fig. S10). In a few species (RSF2, RSF7, and RSF8), a lower spatial structure (*F*_ST_ < 0.2) of most alleles between waterworks was observed (Fig. S10). These observations differ from those on soil bacterial populations across a meadow obtained using the same approach, where most populations had mean gene *F*_ST_ values <0.05 (31). These contrasting results are consistent with a higher physical separation across the set of waterworks than across samples extracted from a single meadow, where dispersal is more likely to occur.

10.1128/mSystems.01139-21.9 FIG S9 Relationship between waterworks dissimilarity based on major allele frequency of common SNPs and the geographic distance of the waterworks. The dissimilarities between populations in pairs of waterworks are calculated using the Jaccard index from a matrix of major allele frequencies for common SPNs across the waterworks: the value 0 means that the two populations in the two waterworks have the same allele profile. The Mantel test was used to test the strength and significance of correlations (*R*^2^, Mantel statistic *r*; *, *P* < 0.05; **, *P* < 0.01). Blue line shows the linear regression with shadowed region indicating 95% confidence intervals for the slope. The order of the plots from left to right and top to bottom corresponds to RSF1, RSF2, RSF3, RSF5, RSF6, RSF7, RSF8, and RSF13. Download FIG S9, PDF file, 0.7 MB. Copyright © 2022 Palomo et al. 2022 Palomo et al. https://creativecommons.org/licenses/by/4.0/ This content is distributed under the terms of the Creative Commons Attribution 4.0 International license.

10.1128/mSystems.01139-21.10 FIG S10 Boxplot showing the *F*_ST_ values for each species (each dot represents the *F*_ST_ value measured as the differences in allele frequencies between populations of the same species found in two distinct waterworks). Only species with more than two data points are shown. Differences between the mean *F*_ST_ were assessed by a Dunn’s test; the same letter have means not significantly different from each other (*P* < 0.01). Download FIG S10, PDF file, 0.1 MB. Copyright © 2022 Palomo et al. 2022 Palomo et al. https://creativecommons.org/licenses/by/4.0/ This content is distributed under the terms of the Creative Commons Attribution 4.0 International license.

We also investigated local regions of the *Nitrospira* genomes with significantly higher F*_ST_* values, as this is characteristic of local (here, in each waterworks) selective pressures (31). Twelve loci with unusually high F*_ST_* were found in six of the *Nitrospira* populations (Fig. S11 at https://figshare.com/articles/figure/Fig_S11/17099036 and Table S8 at https://figshare.com/articles/dataset/Table_S8/17102996), one of them containing genes involved in nitrogen assimilation (*Nitrospira* sp. RSF2) (Table S8 at https://figshare.com/articles/dataset/Table_S8/17102996). However, only two of these loci with unusually high site-specific differentiation of alleles (high *F*_ST_) also had few recombinant events and low nucleotide diversity (Table S8 at https://figshare.com/articles/dataset/Table_S8/17102996), which signal recent selective sweeps (31). These results suggest that, contrary to what has been observed in several natural populations (31–34), gene-specific sweeps seem to play a minor role in the evolution of *Nitrospira* species inhabiting the waterworks. A possible explanation could be that the low recombination rate that characterizes the waterworks *Nitrospira* populations (Fig. 3B, discussed below) limits the possibility of gene-specific sweeps (35).

Overall, across the 12 waterworks, all species presented significant genomic microdiversity, but this diversity was not always represented locally, with a few occurrences of patterns consistent with clonal expansion. The reason for the difference of within-species composition across waterworks is unknown, but the allopatric nature of the communities likely contributes to their persistence.

### Evolutionary processes at the whole-genome level.

The *Nitrospira* populations were characterized by a low degree of homologous recombination as indicated by the consistently high value of linkage disequilibrium (*D*′) of their genomes (Fig. 3B), which measures the nonrandom association of alleles at two loci (*D*′ is only <1 if all possible combinations of a pair of biallelic sites are observed [36]; lower *D*′ values indicate higher levels of homologous recombination). A similarly low degree of homologous recombination was observed for other abundant non-*Nitrospira* populations of the waterworks (*n* = 12; genome completeness, 92.6% ± 5.5; contamination, 2.1% ± 2.0) (Fig. 3B and Table S6 at https://figshare.com/articles/dataset/Table_S6/17099159). In general, this evolutionary process was lower in the waterworks populations than in populations inhabiting a grassland meadow (Fig. 3B), where a similar analysis was conducted (31). To further examine the relative effect of homologous recombination on the genetic diversification of the populations, we measured the rates at which nucleotides become substituted as a result of recombination versus mutation (*r/m*). Most of the *Nitrospira* populations had a relatively low *r/m* (*r/m *< 2) compared to recombinogenic species reported in literature (*r/m *> 4) (37) (Fig. S12 at https://figshare.com/articles/figure/Fig_S12/17099099), although in one case (RSF15_CG24_B), the rate was similar to the value reported for a Streptomyces flavogriseus population (*r/m *= 28) considered to be approaching panmixia (38). Overall, these results suggest a low effect of recombination in the populations of the waterworks. An increasing recombination rate has been associated with fluctuating environments as a source of variation which can accelerate adaptation favoring survival in this type of environment (39, 40). On the other hand, constant environments, such as the waterworks studied here, tend to reduce the recombination rate of their residents (39).

The *Nitrospira* populations were characterized by strong purifying selection. We used the relation between nonsynonymous and synonymous polymorphisms (pN/pS) to investigate this evolutionary process. We detected pN/pS of <1, indicating purifying selection, for all *Nitrospira* species (Fig. 3C). Similar results were observed for other abundant populations of the waterworks (Fig. 3C). Purifying selection has frequently been observed in populations in the environment, and it was the case for populations in a grassland meadow (31) (pN/pS, 0.56 ± 0.17; *n* = 19), but this process seems to be especially strong in the waterworks populations (pN/pS, 0.34 ± 0.05; *n* = 24) (two-sample *t* test, *P* < 0.0001) (Fig. 3C). This suggests that their genomes could have reached an adaptive optimum for this stable environment, which is maintained by purging nonsynonymous mutations.

**FIG 4:**
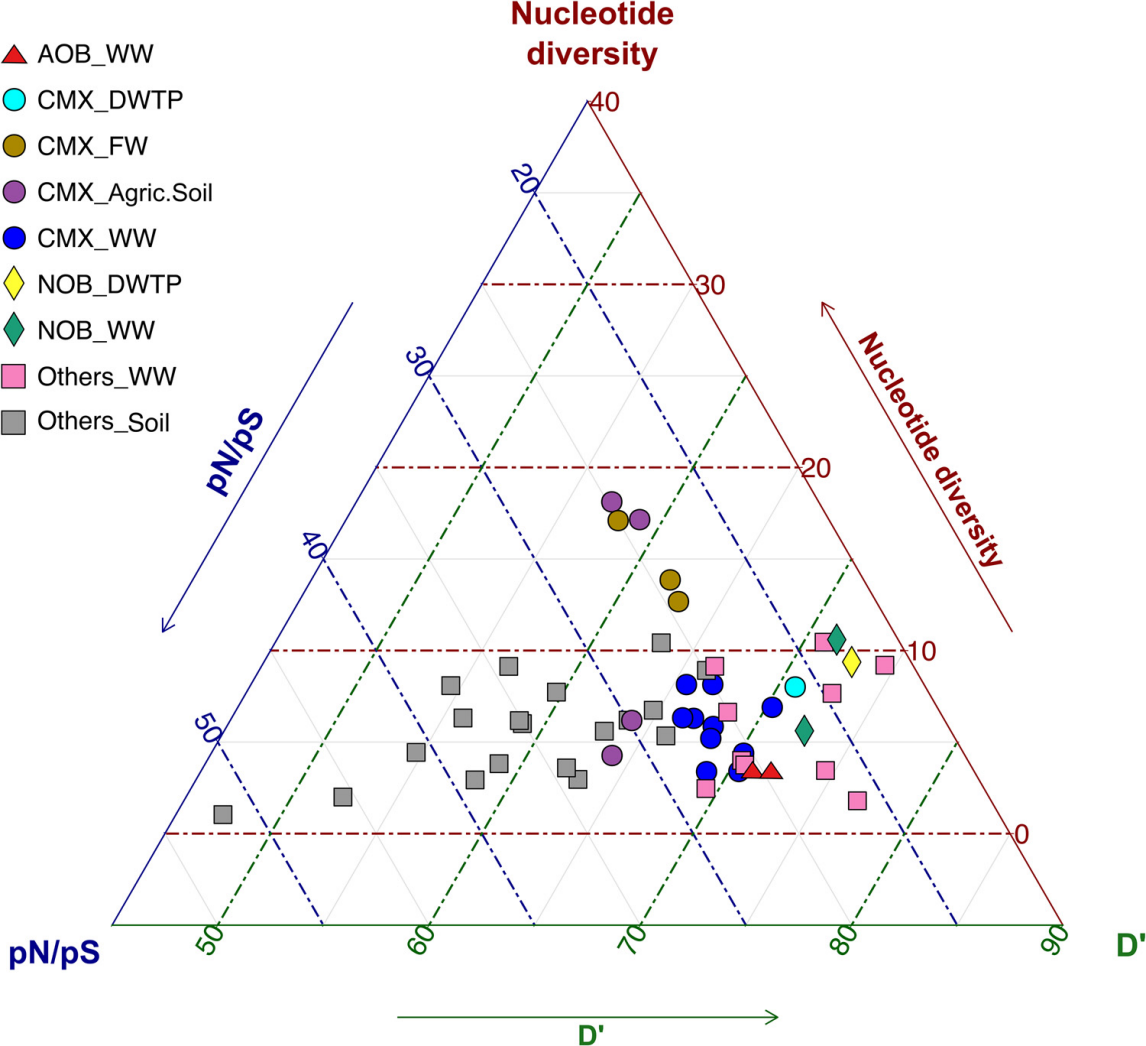
Impact of environment and microbial type in evolutionary metrics. Triplot composed of the nucleotide diversity, pN/pS ratio, and *D*′ values for the bacterial populations of this study’s waterworks (WW), the most abundant bacterial populations across a grassland meadow (38) (Soil) and other abundant *Nitrospira* populations recovered from other systems. FW, freshwater; DWTP, drinking water treatment plan; Agric.Soil, agricultural soil). The data reported in this table (including the MAG IDs) can be found at https://figshare.com/articles/dataset/Table_S6/17099159.

Interestingly, the degree of recombination and diversity across different *Nitrospira* populations varied substantially with habitat (Fig. 4). High variability of recombination in closely related bacterial species has occasionally been reported (41), and lifestyle appears as one of the most important factors to explain this variability (16, 41). Our analysis of *Nitrospira* populations from different habitats (drinking water treatment plants [DWTP], freshwaters, and soils) suggests that the environment also influences evolutionary processes in free-living bacteria: different bacterial types in the same environment tended to share similar features (Fig. 4), while the evolutionary characteristics of comammox *Nitrospira* populations differed depending on the environment from where they were retrieved (Fig. 4). Comammox species in the studied waterworks and other DWTP were characterized by low recombination, strong purifying selection, and moderate microdiversity (Fig. 4). In contrast, comammox from freshwater and soils had higher microdiversity and, especially, recombination rate (Fig. 4 and Table S6 at https://figshare.com/articles/dataset/Table_S6/17099159). Intriguingly, we consistently observed that canonical *Nitrospira* species showed features similar to those of comammox *Nitrospira* but with even stronger purifying selection (Fig. 4). This feature, together with the much lower richness observed in canonical *Nitrospira* than comammox bacteria (Fig. 2), suggests that competition can play a more intense role in canonical *Nitrospira*, which might select for few species optimally adapted to this type of stable environment. However, a broader analysis is required to confirm this hypothesis.

### Evolutionary processes at the gene level.

**FIG 5:**
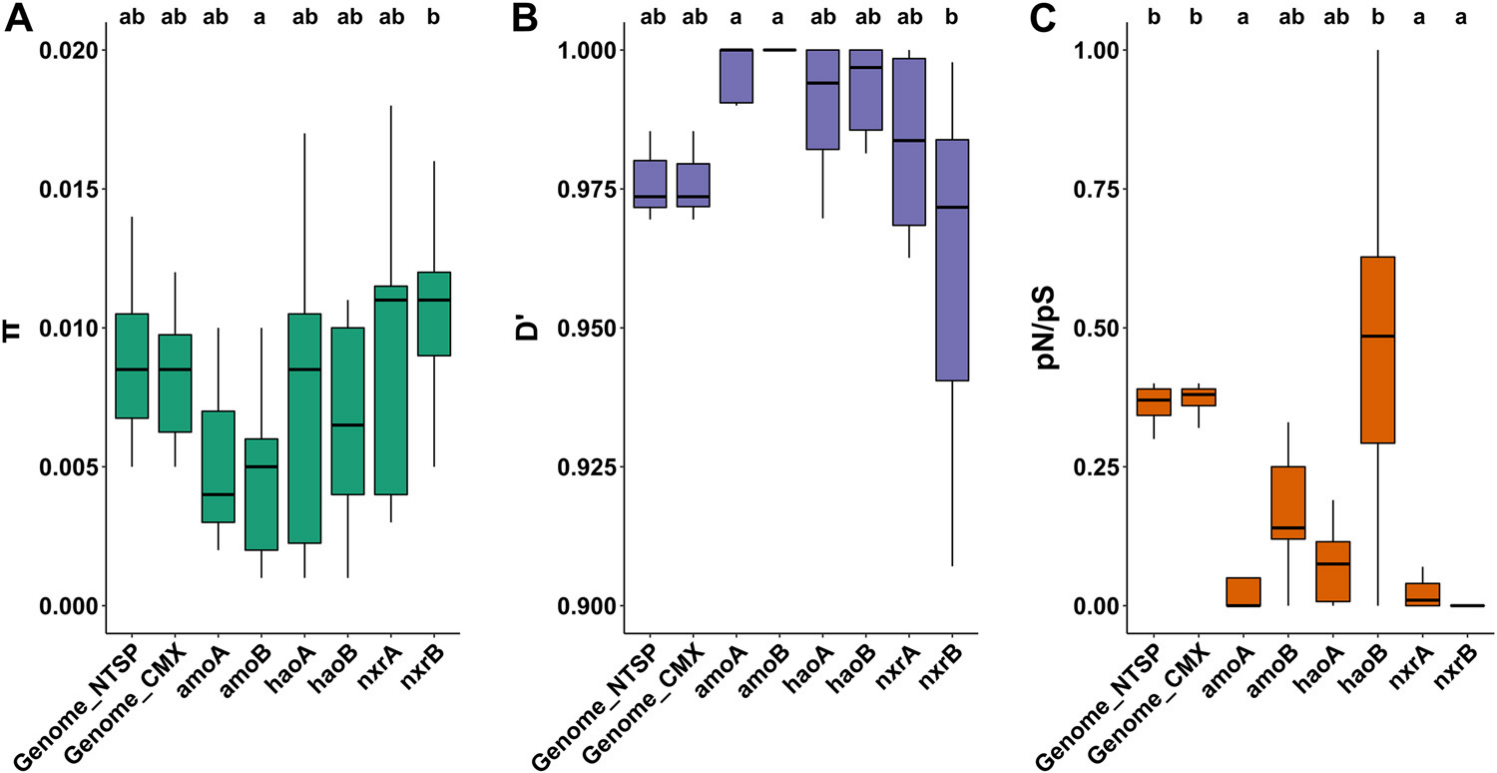
Evolutionary metrics of nitrification genes in *Nitrospira* populations across 12 waterworks. (A) Boxplot of nucleotide diversity of *Nitrospira* bacterial populations for whole genome (all *Nitrospira* and comammox *Nitrospira*) and nitrification genes. Differences between the mean nucleotide diversities were assessed by a Dunn’s test; the same letter means no significant difference (*P* > 0.05). (B) Boxplot of linkage disequilibrium of *Nitrospira* bacterial populations for whole genome (all *Nitrospira* and comammox *Nitrospira*) and nitrification genes. Differences between the mean linkage disequilibriums were assessed by a Dunn’s test; the same letter means no significant difference (*P* > 0.05). (C) Boxplot of pN/pS ratios of *Nitrospira* bacterial populations for whole genome (all *Nitrospira* and comammox *Nitrospira*) and nitrification genes. Differences between the mean pN/pS ratios were assessed by a Dunn’s test; the same letter means no significant difference (*P* > 0.01).

In addition to a genome-wide analysis, we investigated the evolutionary processes at the gene level. In the studied *Nitrospira* populations, genes involved in nitrification (ammonia monooxygenase, *amoA* and *amoB*; hydroxylamine dehydrogenase, *haoA* and *haoB*; nitrite oxidoreductase, *nxrA* and *nxrB*) generally had a similar nucleotide diversity (Fig. 5A) and homologous recombination rate (*D*′) (Fig. 5B) compared to the rest of the genome, but with higher levels of purifying selection (pN/pS) (Fig. 5C). The nucleotide diversities of genes related to nitrification were very similar, with the exception of *amoB*, which had a significantly lower nucleotide diversity than *nxrB* (*P* < 0.05) (Fig. 5A). A similar pattern was detected for the recombination, but in this case, *amoA*, as well as *amoB*, had significantly lower recombination than *nxrB* (*P* < 0.05) (Fig. 5B). We observed a very strong purifying selection for most of the nitrifying genes, especially for *amoA*, *nxrA*, and *nxrB* (*P* < 0.01) (Fig. 5C). In the case of *nxrB*, not a single nonsynonymous mutation was found in most *Nitrospira* species (0 to 1 nonsynonymous site versus 17 to 66 synonymous sites), even though this gene had a higher nucleotide diversity and homologous recombination (Fig. 5A and B). Our observations on selection are in line with previous studies, as, generally, essential genes and enzymes catalyzing reactions that are difficult to bypass through alternative pathways are subject to higher purifying selection than nonessential ones (42–45).

Even though the average pN/pS values were below 1 in all *Nitrospira* species (Fig. 3), indicating purifying selection, genes with pN/pS values above 1 and significantly higher than the genomic average were detected in each species (Fig. S13 at https://figshare.com/articles/figure/Fig_S13/17099114). Many of those genes under positive selection were related to defense mechanisms against phages (e.g., genes putatively involved in phage entry into cells, ribonucleases, genes coding proteins associated with restriction-modification systems, and genes related to toxin-antitoxin systems) (Table S9 at https://figshare.com/articles/dataset/Table_S9/17102999). Comparable findings were made in other abundant species from the waterworks (Table S10 at https://figshare.com/articles/dataset/Table_S10/17103002), in the additional *Nitrospira* populations retrieved from other environments (Table S10 at https://figshare.com/articles/dataset/Table_S10/17103002), as well as in E. coli and *Vibrio* sp. strains, respectively (46, 47). These observations suggest that positive selection in phage defense-related genes is widespread across bacteria and highlights the evolutionary arms race occurring between phages and bacteria as an important driver in bacterial ecology and evolution (35, 48, 49). Additionally, we found nondefense mobile genetic elements, such as transposons and integrases, with significantly higher pN/pS values than the genome average in the *Nitrospira* spp. (Table S11 at https://figshare.com/articles/dataset/Table_S11/17103008).

### Conclusions.

A major unresolved question is how the relationship between ecology and evolution shapes complex communities in the environments. Here, we used a model microbial system with relatively stable conditions to examine this question. By analyzing the microbial communities in 12 groundwater-fed rapid sand filters, we observed that co-occurring comammox *Nitrospira* spp. dominate all sites, but with local differences in species composition. This suggests that the abundant comammox *Nitrospira* spp. exploit slightly different niches, which could partially be explained by the inlet water chemistry composition and their different genetic repertoires. At the subspecies level, comammox *Nitrospira* spp. are characterized by strong purifying selection and low recombination. These features, together with the occasional genome sweeps we detected, suggest that the subspecies possibly occupy a narrow niche to which they are optimally adapted. In contrast, we also showed that the relative magnitude of these evolutionary processes was different in comammox *Nitrospira* in habitats where environmental conditions are less stable and immigration more intense. Thus, we conclude that the evolutionary processes that drive the diversification of *Nitrospira* are dependent on the environment, as opposed to intrinsic properties of the species.

## MATERIALS AND METHODS

### Sampling, sequencing, and metagenomic assembled genomes recovery.

The sampling description, DNA extraction, and sequencing have been previously described (21). Briefly, filter material was collected from two locations at the top of the filters of 12 Danish waterworks. DNA was extracted from 0.5 g of sand material using the MP FastDNA Spin kit (MP Biomedicals LLC, Solon, OH, USA). DNA libraries were generated using the 24 extracted DNA with the Nextera XT DNA library preparation kit (Illumina Inc.) according to the manufacturer’s instructions. Samples were sequenced in one lane, with 2 × 150 paired-read sequencing on the Illumina HiSeq4000 at BGI’s facility in Copenhagen. Generated reads were trimmed and filtered, including adapters removal, using Trimmomatic v.0.22 (threshold quality, 15; minimum length, 40) (50). FastQC (Babraham Bioinformatics [http://www.bioinformatics.babraham.ac.uk/projects/fastqc/]) was used to evaluate the quality of the obtained reads. Coassembly of high-quality reads was conducted using IDBA-UD (51) with the options --pre_correction --min_contig 1000. Additionally, single-sample assemblies were performed following the same procedure. As it has been shown that the combination of multiple binning algorithms outperforms the usage of one single algorithm (52), here, we performed metagenomic binning using MetaBAT (53), MaxBin2 (54), CONCOCT (55), MyCC (56), BinSanity (57), and COCACOLA (58) (Fig. S1 in the supplemental material). The quality of the resulting genomes was improved using Binning_refiner (59). Simultaneously, we ran the software metaWRAP (60), which also take advantage of multiple binning tools to recover genomes from the coassembly. The generated bins were improved using the refinement module of metaWRAP. Selection of the best genomes recovered with the different binning algorithms was done with DAS Tool (61). dRep (62) was applied to dereplicate all the selected best bins with the secondary clustering threshold set at 99% genome-wide average nucleotide identity (gANI). On the other hand, mmgenome (63) was applied to the single-sample assemblies to recover *Nitrospira* genomes following the strategy described elsewhere (13). The generated *Nitrospira* genomes were combined with the dereplicated bins and subjected to the reassembly module of metaWRAP with the aim of improving the quality of the bins. dRep at 99% gANI was used again for dereplication. RefineM (--cov_corr = 0.8) (64) was used to refine the resulting dereplicated bins by removing scaffolds with divergent GC content or tetranucleotide frequencies. Furthermore, the binning and refinement modules from metaWRAP were applied to the coassembly of the six RSF samples used in Palomo et al. (5). Obtained bins, together with the reported ones in Palomo et al. (5, 13), were dereplicated using dRep at 99% gANI. A refinement step with RefineM was applied on the resulting dereplicated bins. These refined bins, together with the bins recovered from the 12 waterworks of this study, were dereplicated as described above. In addition, the assembly quality of the *Nitrospira* bins was improved by alignment against related complete or draft genomes using the multidraft-based scaffolder (MeDuSa) (65). The overall procedure here described can be visualized in Fig. S1. The completeness and contamination of the bins were evaluated using CheckM (66).

### Species abundance estimation.

A 95% average nucleotide identity (ANI) cutoff was used to define species as proposed by Klappenbach et al. (18). The retrieved MAGs were dereplicated using dRep with the secondary clustering threshold set at 95% gANI. Among the genomes classified as belonging to the same species, the one with higher quality was chosen as representative genome. The species’ abundance and coverage of each representative genome across the metagenomes were assessed using MIDAS (67). Briefly, MIDAS uses reads mapped to 15 universal single-copy gene families (with ability to accurately recruit metagenomic reads to the correct species [67]) to estimate the abundance and coverage of bacterial species from a shotgun metagenome. We used the species retrieved in this study to build the database of universal single-copy genes.

### Genome classification and annotation.

MAGs were classified using the classify workflow of the GTDB-Tk v.0.1.3 tool (68). Open reading frames were predicted using Prodigal v.2.63 (69) and annotated using BLASTP (70) against NCBI nr (71), UniProt (72), KEGG (73), PFAM (74), and eggNOG (75). Genes were assigned to antiphage defense systems using the strategy described in Doron et al. (76).

### Phylogenetic analysis.

Phylogenetic analyses of *Nitrospira* genomes were conducted with the GTDB-Tk v.0.1.3 tool (68, 21) using the *de novo* workflow with a set of 120 single-copy marker proteins and the genome taxonomy database (GTDB) (77). Concatenated alignments were used to construct a maximum-likelihood tree using RAxML v.8.2.11 (78) with 200 rapid bootstraps (determined using the autoMRE option) and the LG likelihood model of amino acid substitution with gamma-distributed rates and fraction of invariant sites (-m PROTGAMMAILGF; best model determined using ProtTest v.3.4.2 [79]). The tree was rooted using two *Leptospirillum* species as outgroup. The rooted tree was visualized using the online web tool from the Interactive Tree of Life (iTol) (80).

### Read mapping, SNP calling, and population genomic analysis.

The population genomic analysis was done following the approach described in Crits-Christoph et al. (31). High-quality reads were mapped to an indexed database of the 176 species MAGs recovered from the waterworks using BWA-MEM (81). The resulting alignments were filtered using SAMtools (82) view -q30 to remove reads with mapping quality less than 30, and also with the script filter_reads.py (31) (with the options -m 96 to retain reads with a percent identity of at least 96% to the reference and -q 2 to ensure uniquely best mapping read pairs in the index). Downstream population genomic analysis was performed on the 24 most abundant species genomes (12 *Nitrospira* ones and 12 other abundant species genomes). For each of these species' genomes, we analyzed data in samples that passed a cutoff of at least 50% of the genome being covered with at least 5× coverage. One hundred forty-nine out of 576 sample genome comparisons (24 genomes × 24 samples) passed this minimum requirement. Sample read mappings were pooled by each waterworks and by all samples across the waterworks. Nucleotide diversity (π), linkage disequilibrium (*D*′), and the ratio of nonsynonymous to synonymous polymorphism (pN/pS) were calculated for each sample, each waterwork, and across all the waterworks as described elsewhere (31) using the scripts provided by the authors. Fixation index (*F*_ST_), which measures the degree of genetic differentiation, was calculated following the same procedure but on sites segregating across two waterworks being compared (for all the possible waterworks comparisons). As Crits-Christoph et al. (31) recommended, only sites with a coverage of at least 20× in each waterworks were used to calculate *F*_ST_. In addition, genes with coverage in a waterworks outside the range of two standard deviations were excluded from the analysis. As previously suggested (31), a two-sample Wilcoxon test was conducted to find out if average linkage of highly differentiated loci differed from the genomic average for each species. Similarly, a two-sample *t* test was used to conclude if average nucleotide diversity of highly differentiated loci differed from the genomic average. Both sets of tests were corrected for multiple hypotheses using the Benjamini-Hochberg method. Recombination to mutation ratio was inferred using mcorr (37).

The strain-level analysis described above was also conducted in other *Nitrospira* MAGs previously recovered (21). Only those ones that passed a cutoff of at least 50% of the genome being covered with at least 5× coverage in any of the metagenomes where the MAGs were present (21) were kept for further analysis.

### Statistical analyses.

All statistical tests were performed using R v.3.5.2 (83). Due to the compositional nature of sequencing data (84), for all statistical analyses, species abundances were analyzed as follows: zeros were replaced with an estimate value using the count zero multiplicative approach with the zCompositions R package (85), and data were further centered log-ratio transformed. *Nitrospira* community dissimilarities were calculated using the Jaccard index. The correlation between the *Nitrospira* community dissimilarities and geographic distances was calculated using the Mantel test (significance obtained after 100,000 permutations). The same analysis was used to assess the correlation between the *Nitrospira* community dissimilarities and the water composition dissimilarity, as well as the correlation between major allele dissimilarities and geographic distances.

Proportionality between abundances of the species across the 24 metagenomes were calculated using the propr R package (86) (with the options metric “rho” “ivar = clr”) and visualized using the corrplot R package (87). For the network analysis, the function getNetwork from propr R package was used to retain proportionalities >0.56 (false-discovery rate [FDR] < 5%). The network was visualized using the igraph R package (88).

Redundancy analysis (RDA) was performed in a stepwise way using the ordiR2step function of the vegan package (89). The analysis was conducted using centered log-ratio-transformed *Nitrospira* species abundances and chemical data of influent water. The constrained ordination model and the variable significance were determined by permutation tests (1,000 permutations) with anova.cca in vegan. Before this analysis, collinearity among explanatory variables was evaluated using variance inflation factor (VIF) in the fmsb package (90). The function vif_func was used to perform a stepwise approach until all variables above a VIF of 5 were excluded (91). Ternary plot was performed using the R package ggtern (92) using the nucleotide diversity, pN/pS ratio, and *D*′ values for the bacterial populations retrieved from the waterworks, as well as most abundant bacterial populations across grassland meadow (31) and other *Nitrospira* populations abundant in other systems (Table S6 at https://figshare.com/articles/dataset/Table_S6/17099159).

Differences between the mean nucleotide diversities of the nitrifying genes, whole *Nitrospira* genomes, and whole comammox *Nitrospira* genomes were assessed using Kruskal-Wallis analysis of variance (ANOVA) followed by Dunn’s test with the Holm-Bonferroni correction. The same analysis was performed for linkage disequilibrium and pN/pS ratios.

### Chemical analysis of influent water.

Ammonium was measured using a standard colorimetric salicylate and hypochlorite method (93), while nitrite was analyzed using a standard method adapted from Grasshoff et al. (94). Nitrate and sulfate were measured by ion chromatography according to American Water Works Association-Water Environment Federation (AWWA-WEF) method 4110 (95). Iron, manganese, and copper were determined by inductively coupled plasma mass spectrometry (ICP-MS) (7700×; Agilent Technologies), while calcium was determined by ICP-optical emission spectrometry (ICP-OES) (Varian, Vista-MPX charge-coupled device [CCD] Simultaneous ICP-OES). Dissolved oxygen and pH were measured with a handheld meter (WTW Multi 3430, with FDO 925 and SenTix 940 probes).

### Data availability.

All raw sequence data and *Nitrospira* genomes retrieved from the Danish rapid sand filters have been deposited at the NCBI BioProject database under accession number PRJNA384587. The rest of the retrieved draft genomes from the Danish rapid sand filters are available on figshare (https://figshare.com/articles/dataset/MAGs_recovered_from_Rapid_Sand_Filters/12962075). Data produced from the strain-level analysis (nucleotide diversity, SPNs, linkage statistics and *F*_ST_ metrics) are available on figshare (https://figshare.com/projects/Evolutionary_ecology_of_natural_comammox_Nitrospira_populations/91217).
