# Supplementary figures and images for: Evolutionary Ecology of Natural Comammox Nitrospira Populations
Source: mSystems. 2022 Jan 11;7(1):e01139-21. doi: 10.1128/msystems.01139-21 (PMC8751384; doi:10.1128/msystems.01139-21)

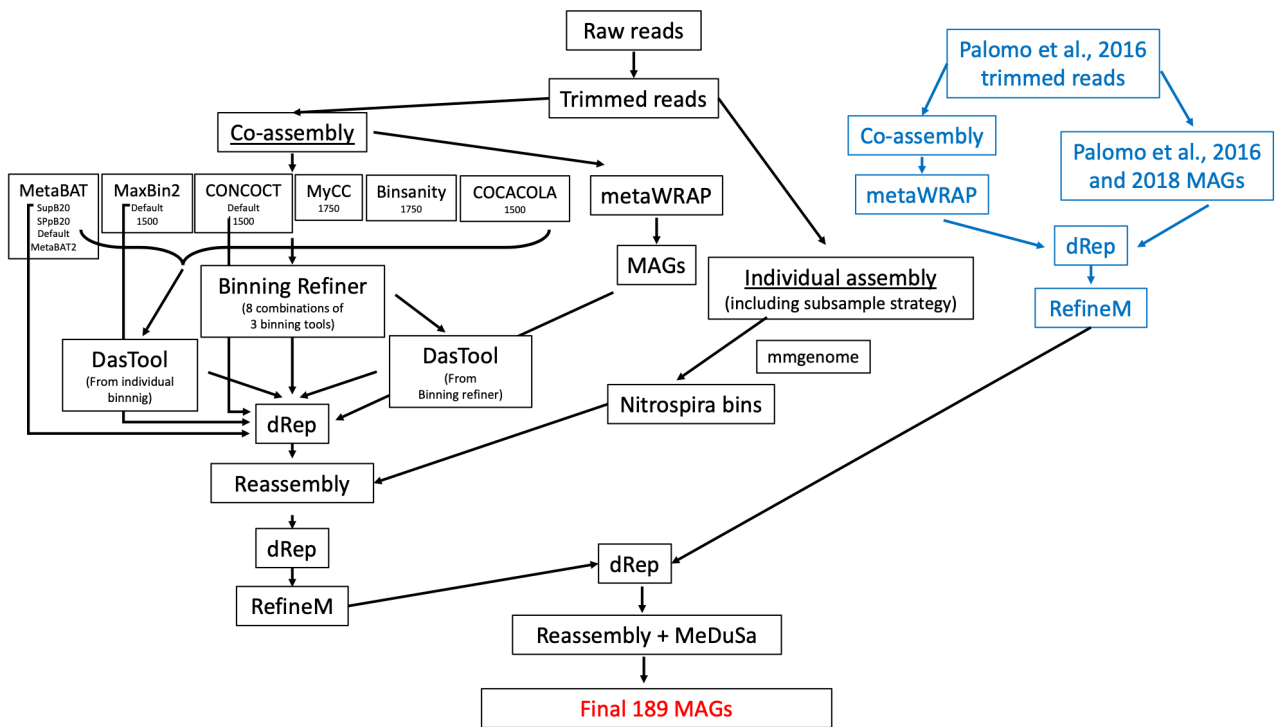

Supplement: FIG S1 [file msystems.01139-21-sf001.pdf]

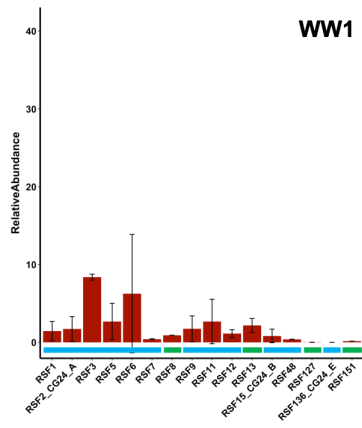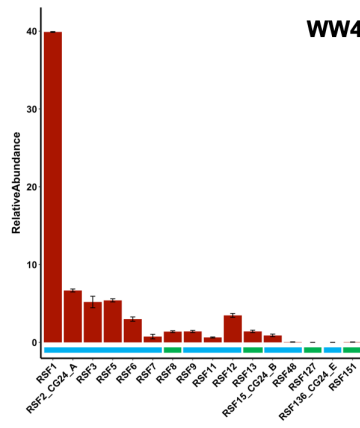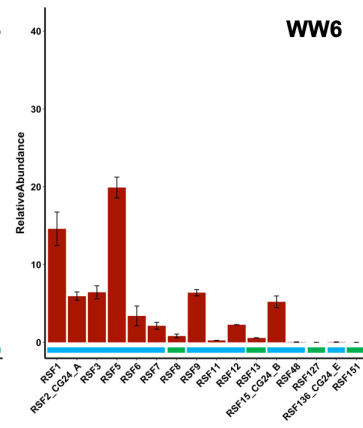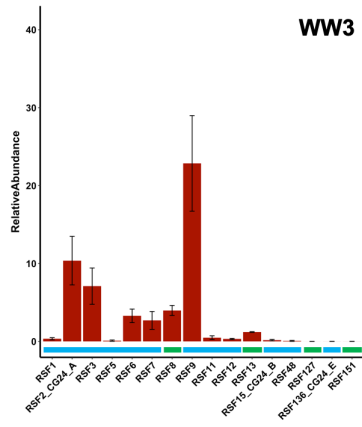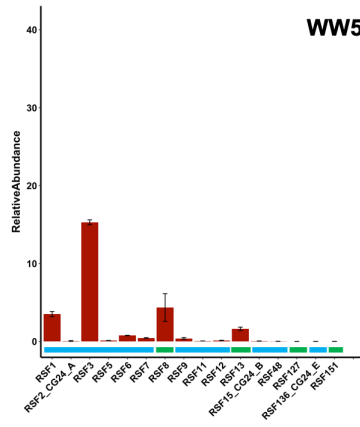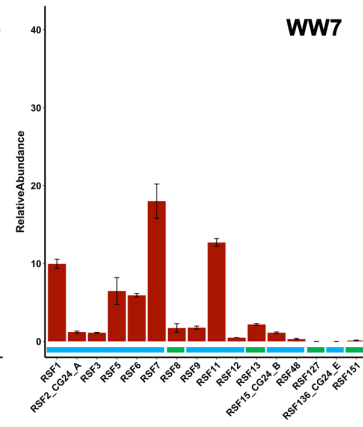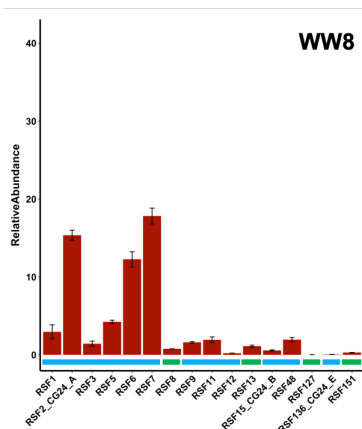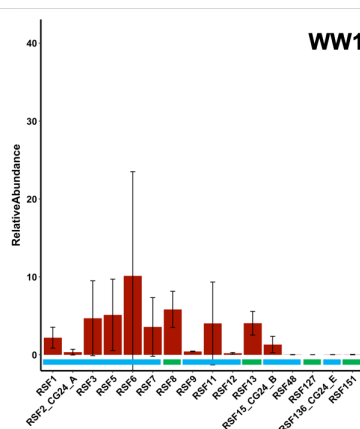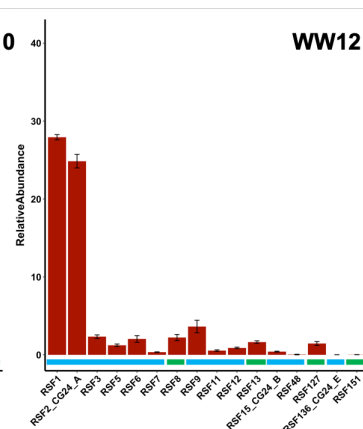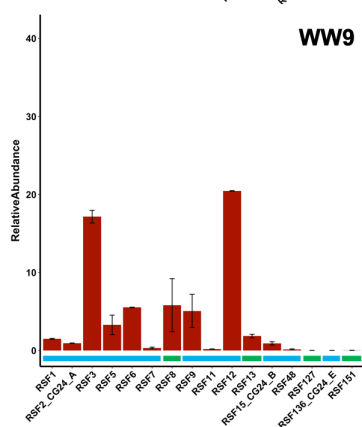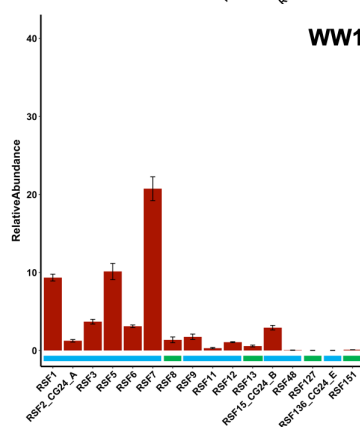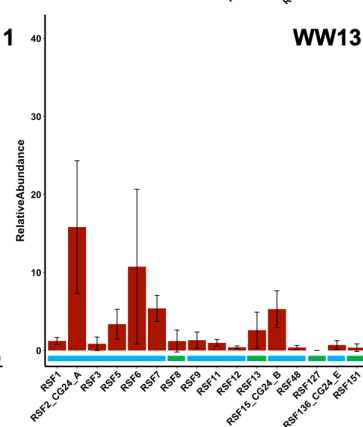

Supplement: FIG S2 [file msystems.01139-21-sf002.pdf]

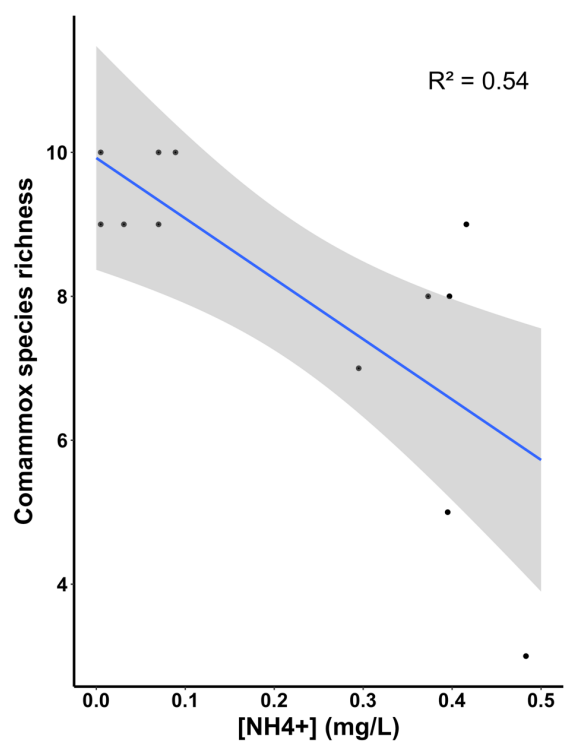

Supplement: FIG S3 [file msystems.01139-21-sf003.pdf]

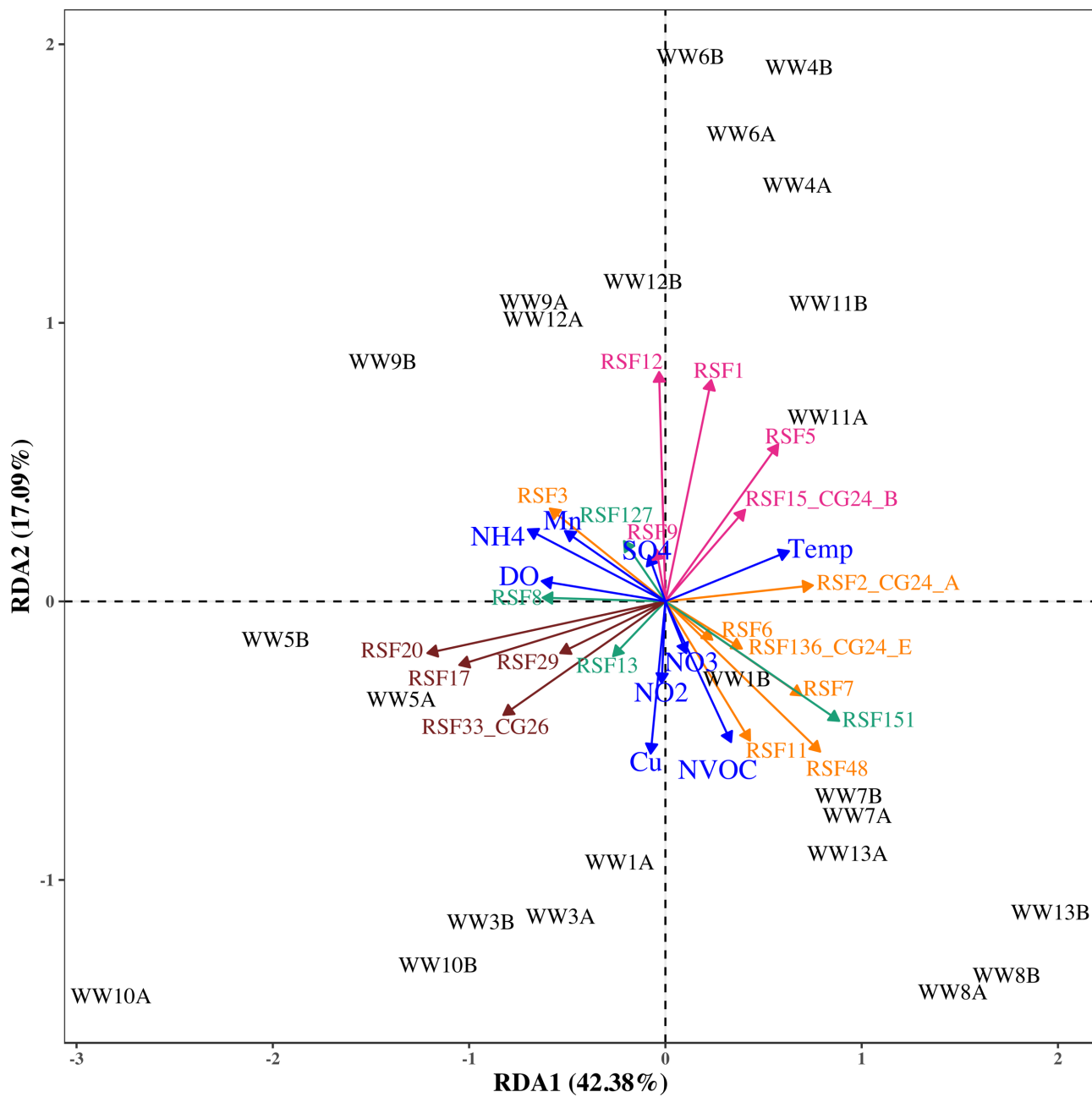

Supplement: FIG S4 [file msystems.01139-21-sf004.pdf]

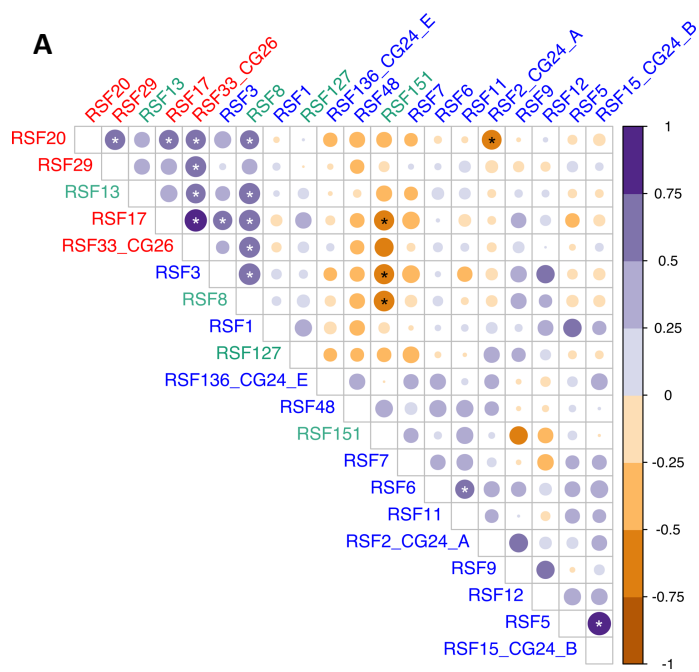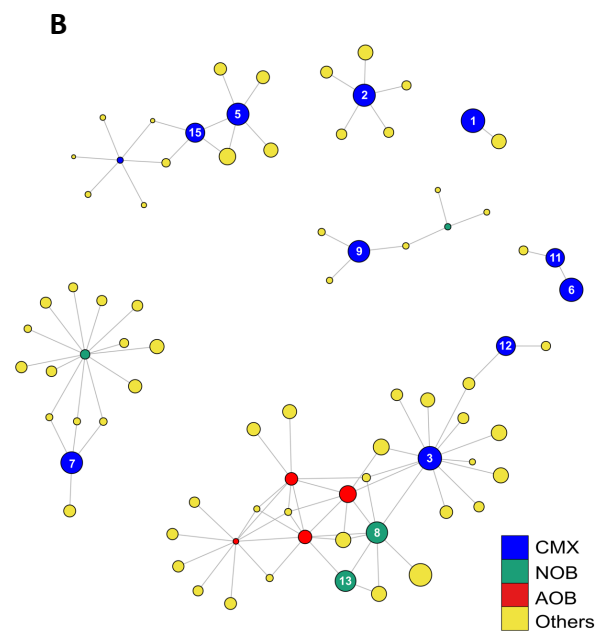

Supplement: FIG S5 [file msystems.01139-21-sf005.pdf]

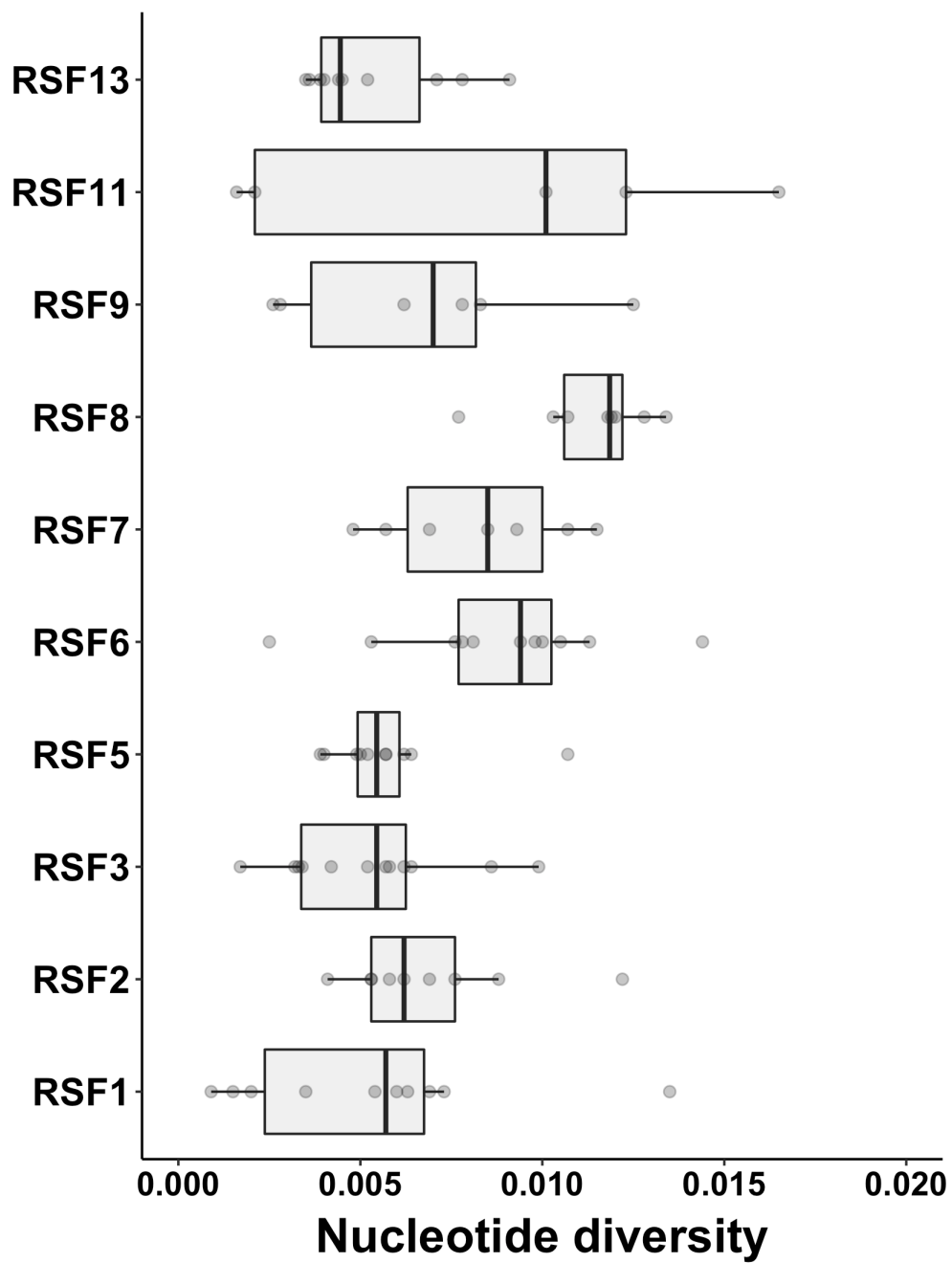

Supplement: FIG S6 [file msystems.01139-21-sf006.pdf]

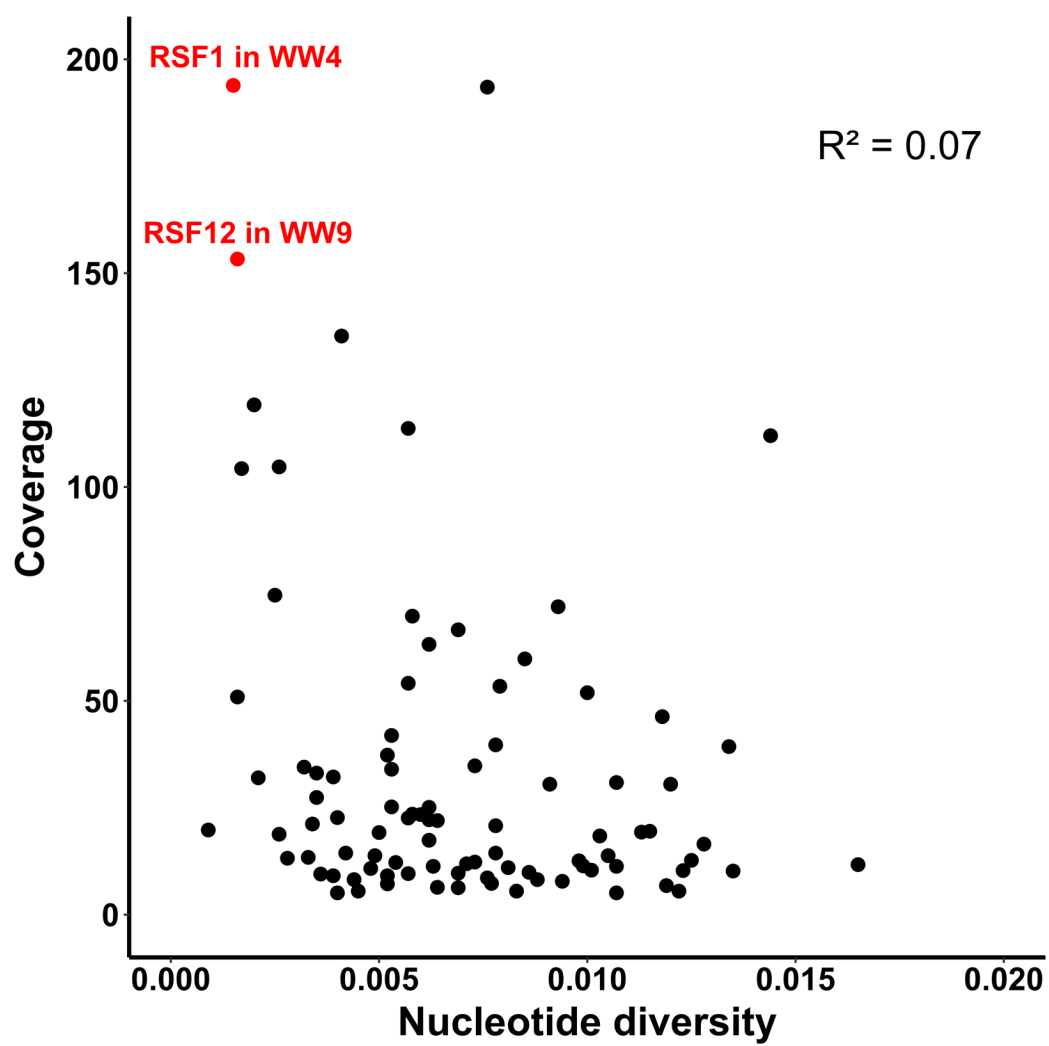

Supplement: FIG S7 [file msystems.01139-21-sf007.pdf]

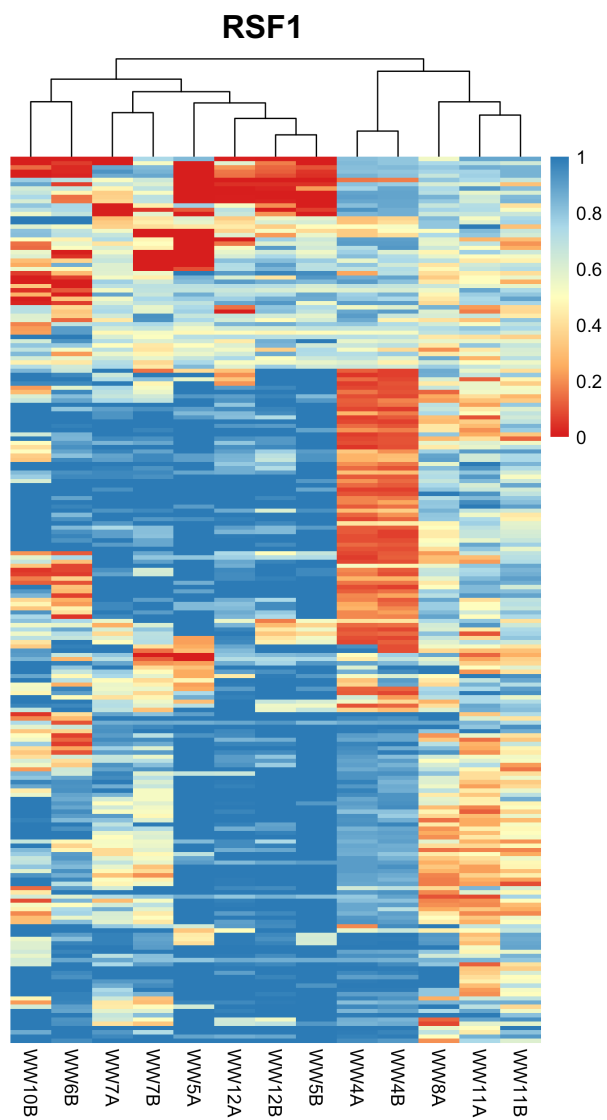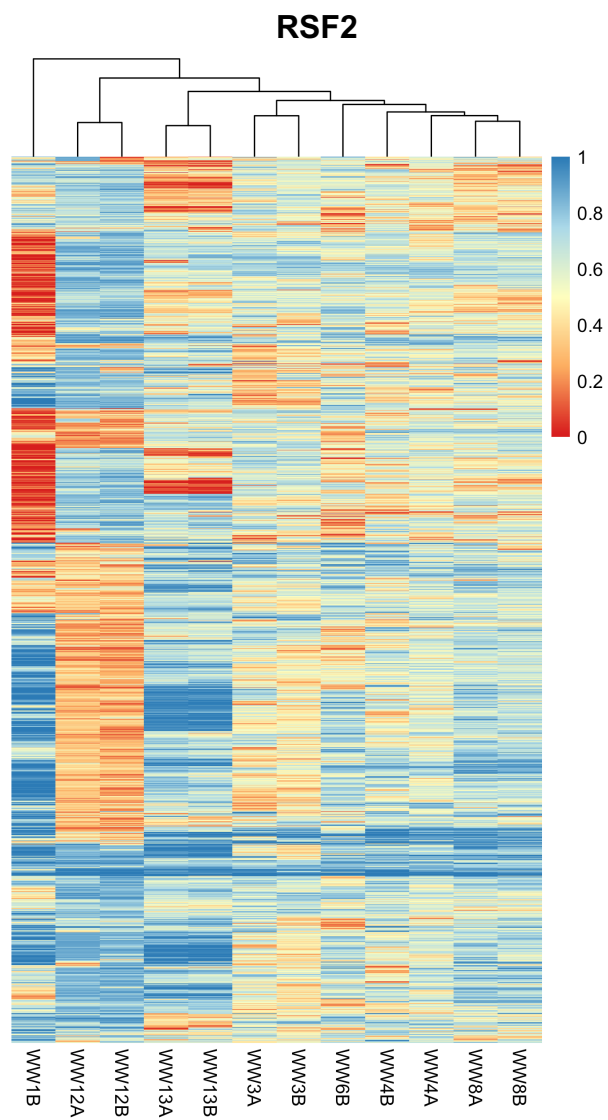

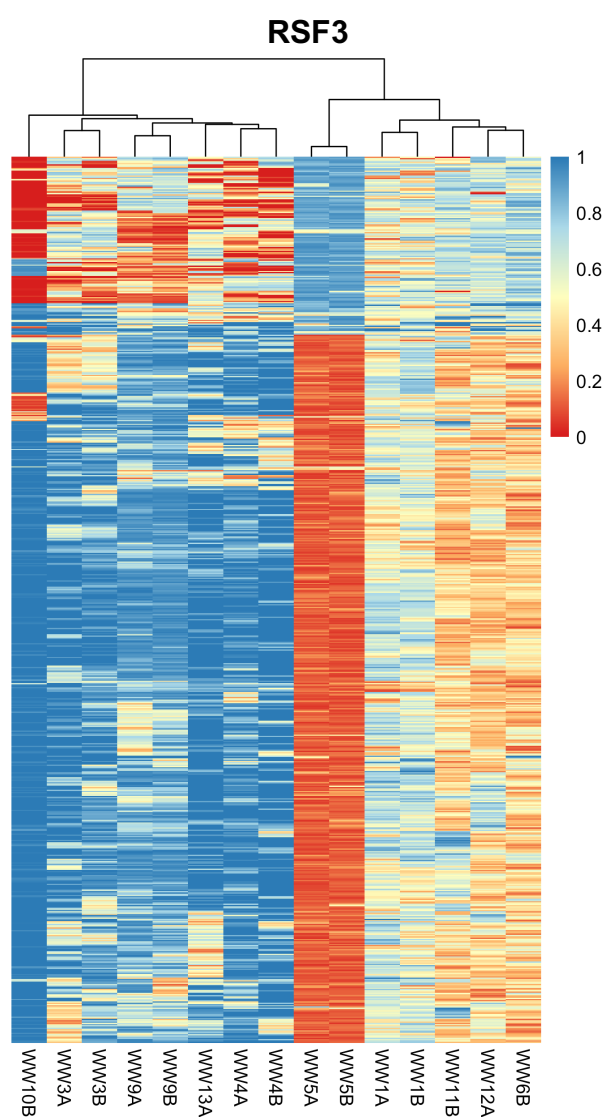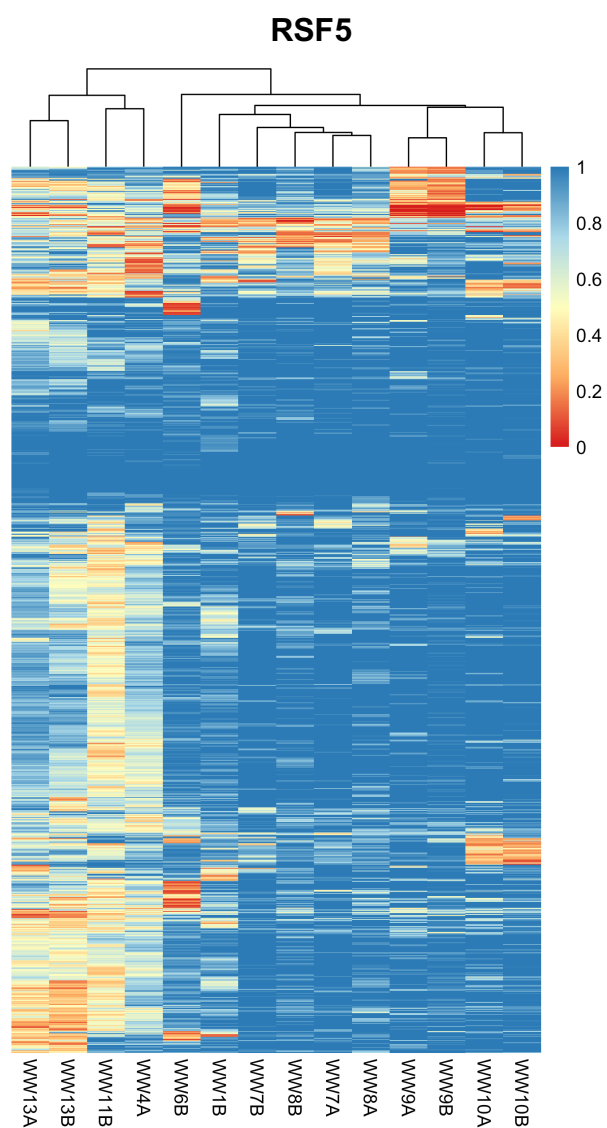

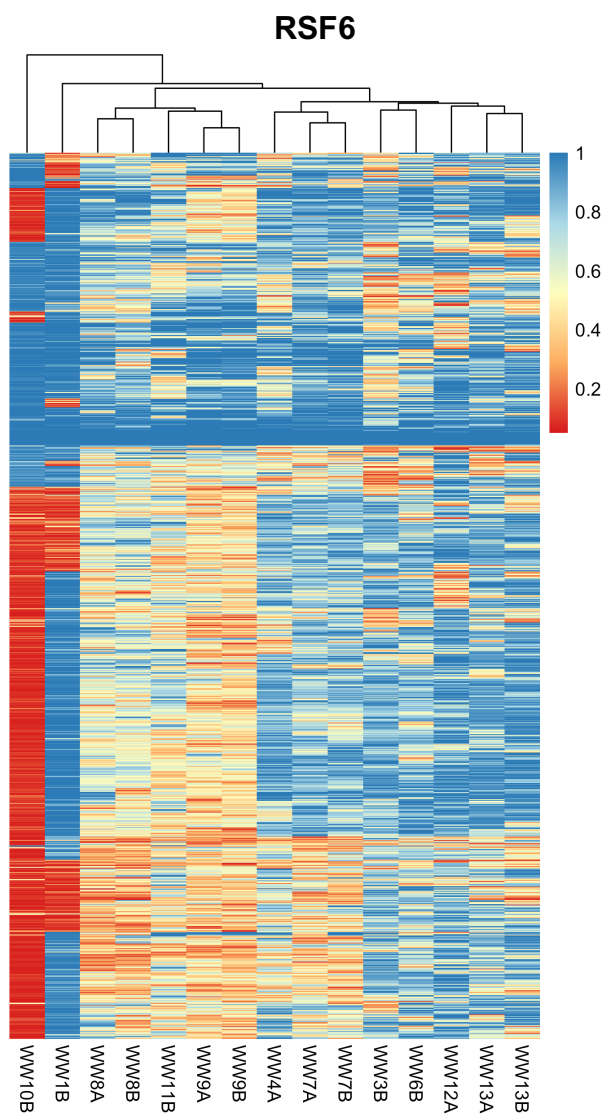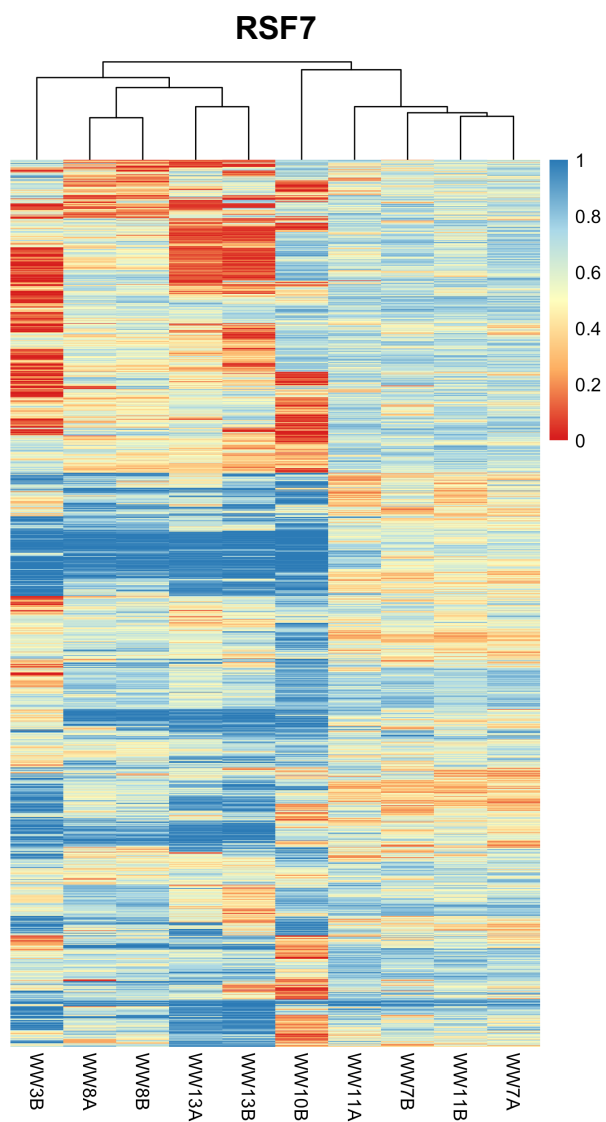

RSF8

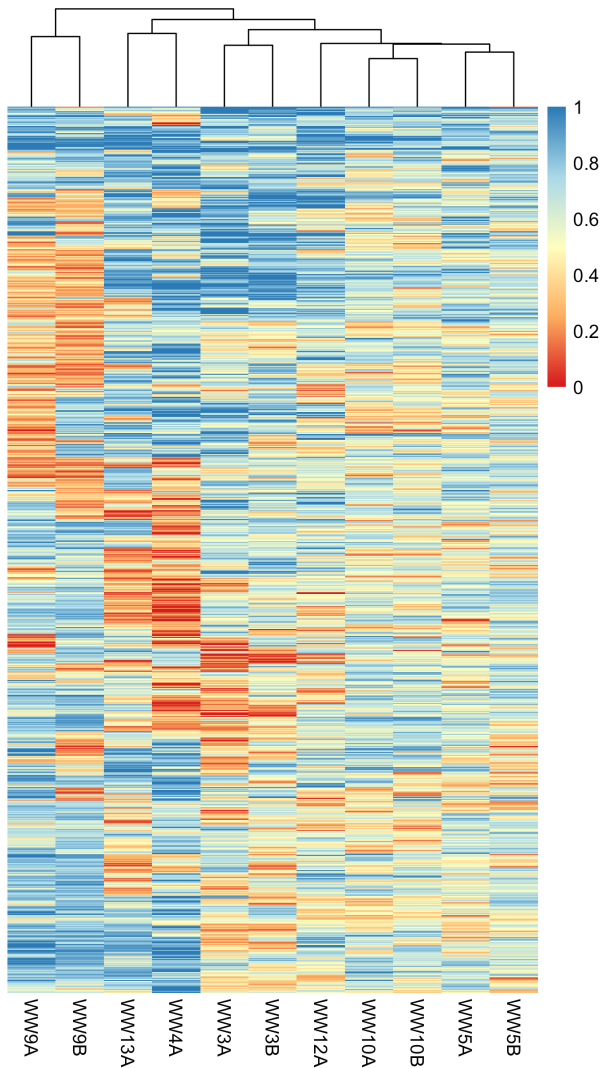

RSF13

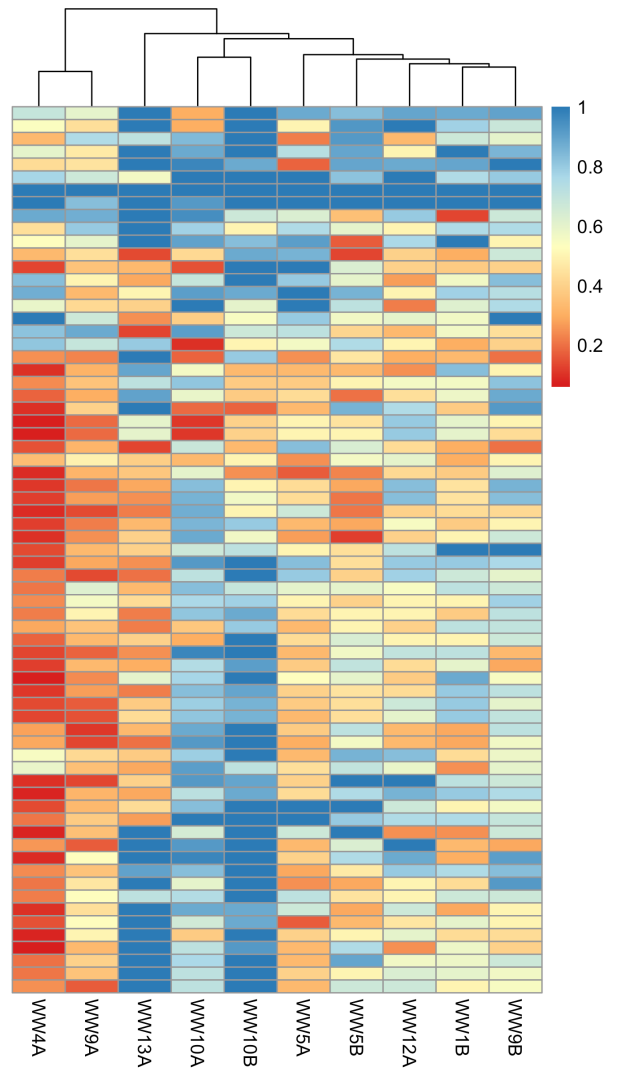

Supplement: FIG S8 [file msystems.01139-21-sf008.pdf]

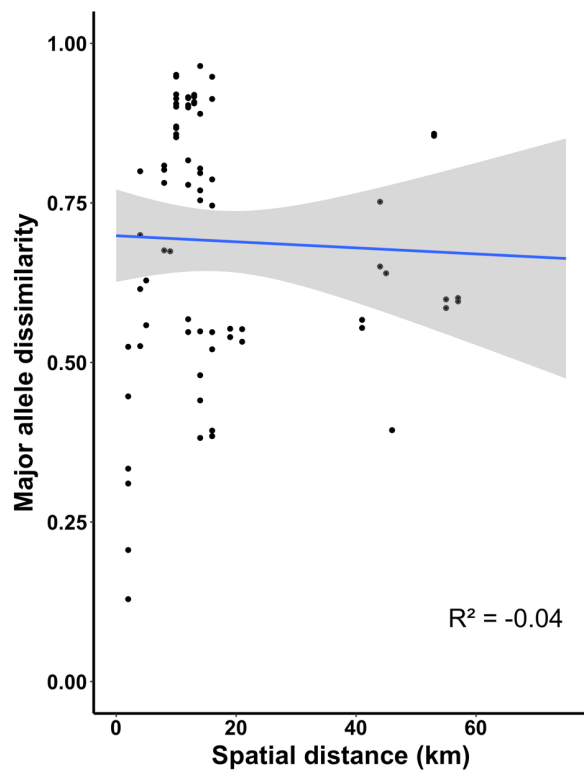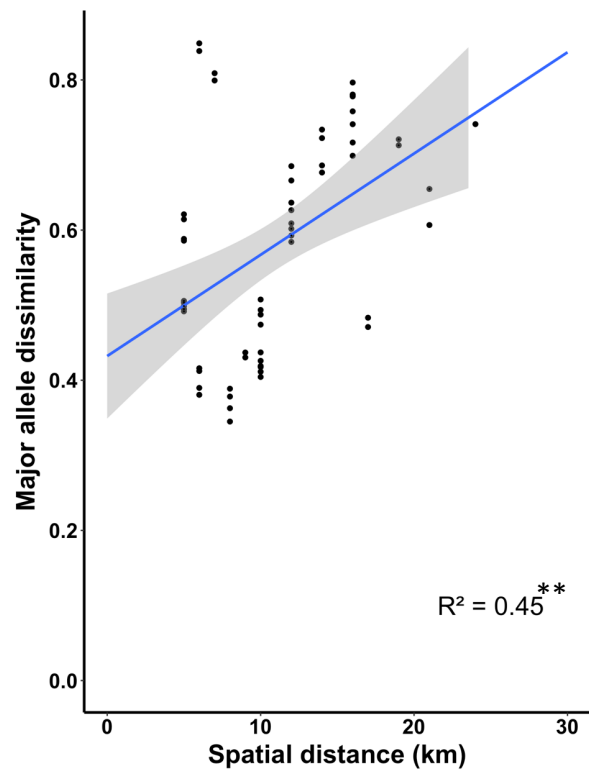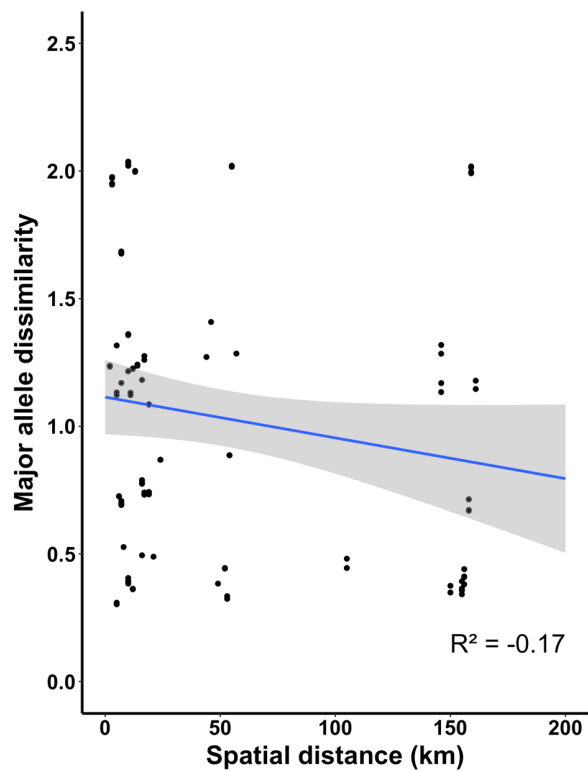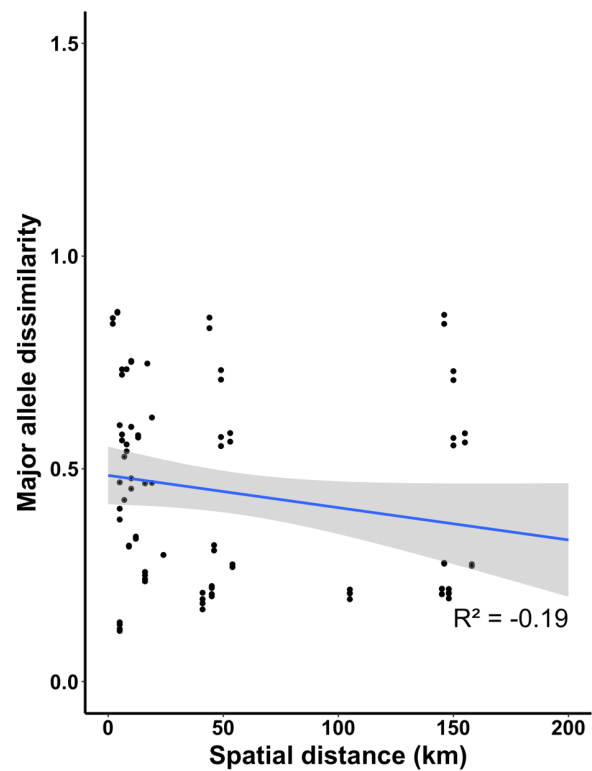

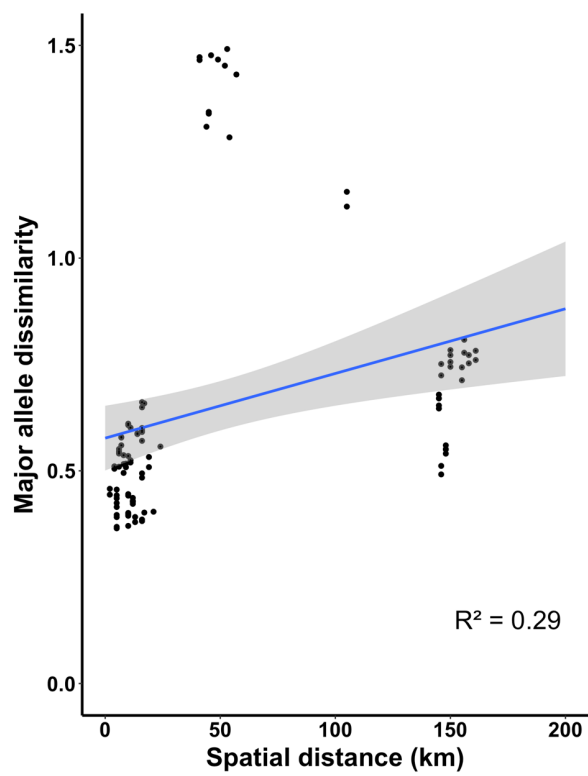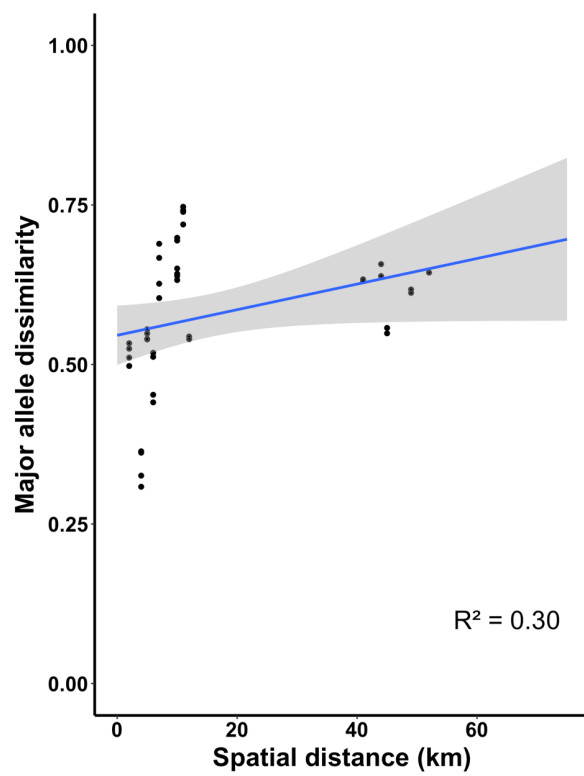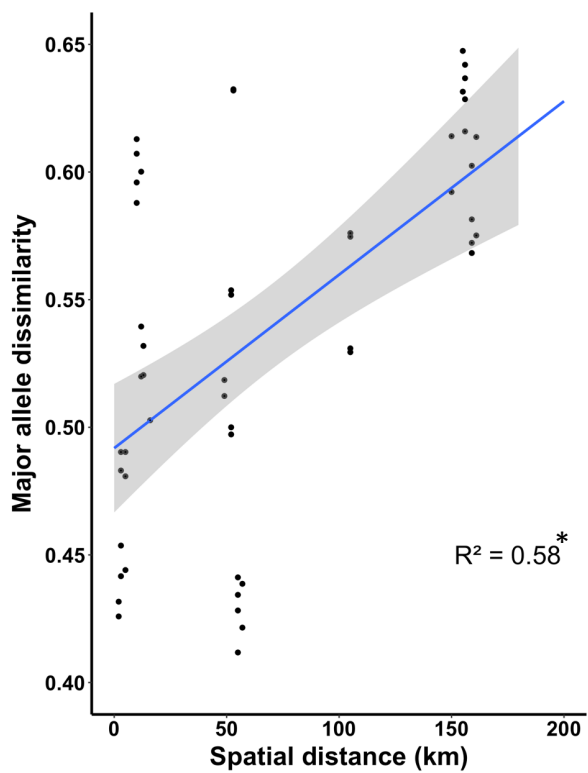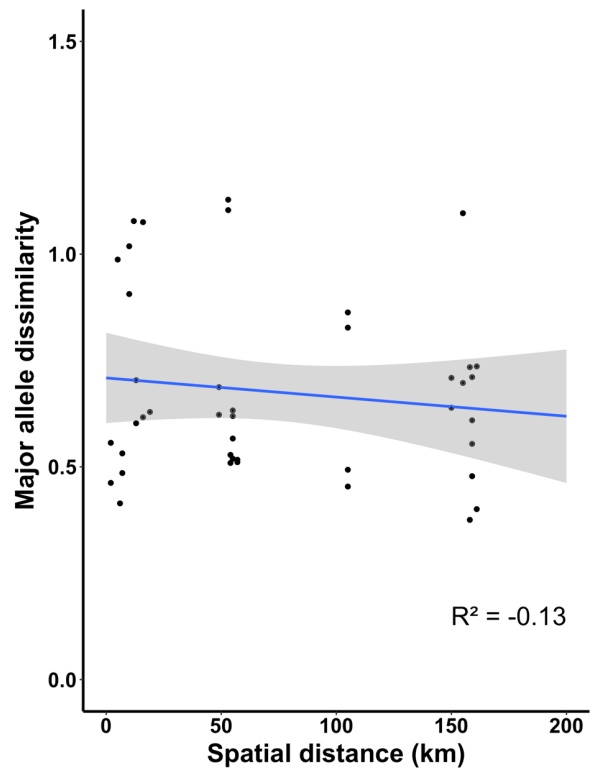

Supplement: FIG S9 [file msystems.01139-21-sf009.pdf]

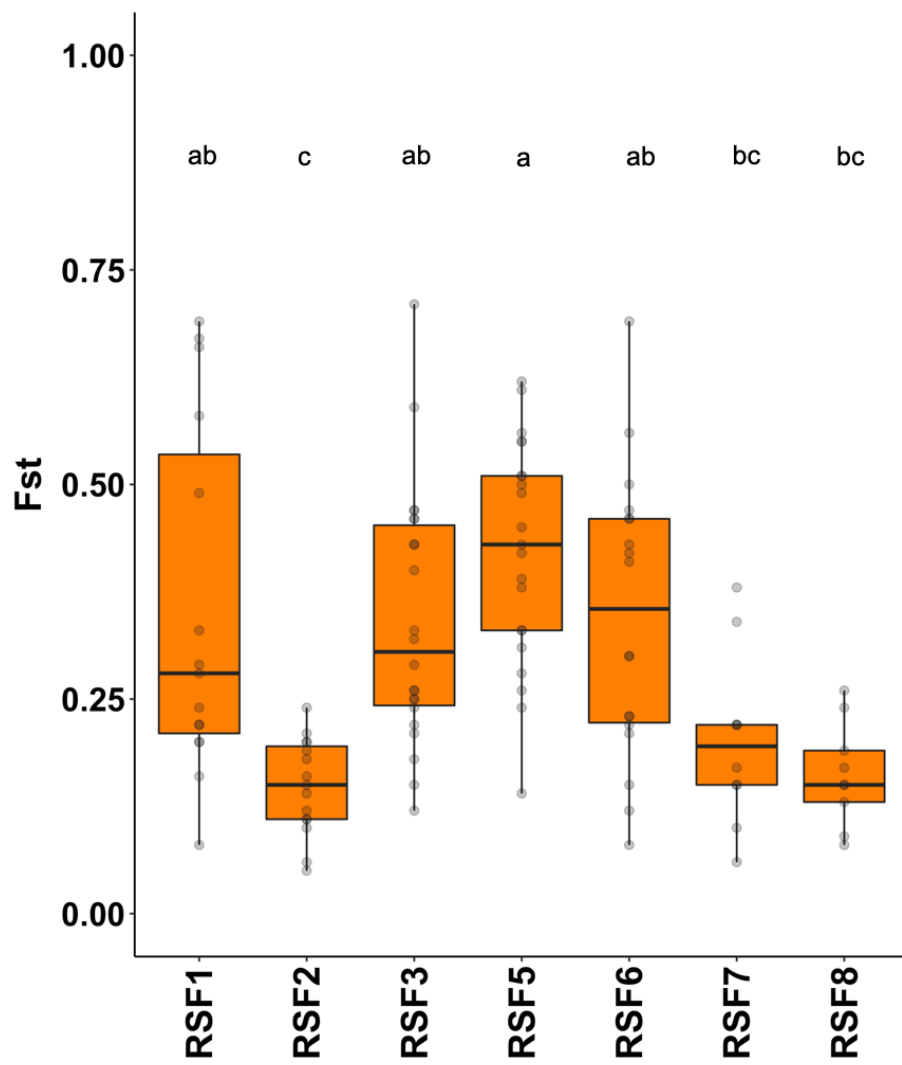

Supplement: FIG S10 [file msystems.01139-21-sf010.pdf]
